# Supporting medication adherence for adults with cystic fibrosis: a randomised feasibility study

**DOI:** 10.1186/s12890-019-0834-6

**Published:** 2019-04-11

**Authors:** Daniel Hind, Sarah J. Drabble, Madelynne A. Arden, Laura Mandefield, Simon Waterhouse, Chin Maguire, Hannah Cantrill, Louisa Robinson, Daniel Beever, Alexander J. Scott, Sam Keating, Marlene Hutchings, Judy Bradley, Julia Nightingale, Mark I. Allenby, Jane Dewar, Pauline Whelan, John Ainsworth, Stephen J. Walters, Alicia O’Cathain, Martin J. Wildman

**Affiliations:** 1 Clinical Trials Research Unit, University of Sheffield, Regent Court, 30 Regent Street, Sheffield, S1 4DA UK; 2 School of Health and Related Research, Regent Court, 30 Regent Street, Sheffield, S1 4DA UK; 3 Centre for Behavioural Science and Applied Psychology, Sheffield Hallam University, Collegiate Crescent, Sheffield, S10 2BQ UK; 4 Sheffield Adult Cystic Fibrosis Unit Sheffield Teaching Hospitals NHS Foundation Trust, Northern General Hospital, Herries Road, Sheffield, S5 7AU UK; 5 Centre for Experimental Medicine, School of Medicine, Dentistry and Biomedical Sciences, Queen’s University, 97 Lisburn Road, Belfast, BT9 7BL UK; 6 Wessex Adult Cystic Fibrosis Service, University Hospital Southampton NHS Foundation Trust, Tremona Road, Southampton, Hampshire SO16 6YD UK; 7 Wolfson Cystic Fibrosis Centre, Nottingham University Hospitals NHS Trust, City Hospital, Hucknall Road, Nottingham, NG5 1PB UK; 8 Health eResearch Centre - Farr Institute, Division of Imaging, Informatics and Data Sciences, School of Health Sciences, Faculty of Biology, Medicine and Health, The University of Manchester, Manchester Academic Health Science Centre, Manchester, UK

## Abstract

**Background:**

Preventative medication reduces hospitalisations in people with cystic fibrosis (PWCF) but adherence is poor. We assessed the feasibility of a randomised controlled trial of a complex intervention, which combines display of real time adherence data and behaviour change techniques.

**Methods:**

*Design:* Pilot, open-label, parallel-group RCT with concurrent semi-structured interviews. *Participants:* PWCF at two Cystic Fibrosis (CF) units. *Eligible:* aged 16 or older; on the CF registry. *Ineligible:* post-lung transplant or on the active list; unable to consent; using dry powder inhalers. *Interventions:* Central randomisation on a 1:1 allocation to: (1) intervention, linking nebuliser use with data recording and transfer capability to a software platform, and behavioural strategies to support self-management delivered by trained interventionists (*n* = 32); or, (2) control, typically face-to-face meetings every 3 months with CF team (*n* = 32). *Outcomes:* RCT feasibility defined as: recruitment of ≥ 48 participants (75% of target) in four months (pilot primary outcome); valid exacerbation data available for ≥ 85% of those randomised (future RCT primary outcome); change in % medication adherence; FEV_1_ percent predicted (key secondaries in future RCT); and perceptions of trial procedures, in semi-structured interviews with intervention (*n* = 14) and control (*n* = 5) participants, interventionists (*n* = 3) and CF team members (*n* = 5).

**Results:**

The pilot trial recruited to target, randomising 33 to intervention and 31 to control in the four-month period, June–September 2016. At study completion (30th April 2017), 60 (94%; Intervention = 32, Control =28) participants contributed good quality exacerbation data (intervention: 35 exacerbations; control: 25 exacerbation). The mean change in adherence and baseline-adjusted FEV_1_ percent predicted were higher in the intervention arm by 10% (95% CI: -5.2 to 25.2) and 5% (95% CI -2 to 12%) respectively. Five serious adverse events occurred, none related to the intervention. The mean change in adherence was 10% (95% CI: -5.2 to 25.2), greater in the intervention arm. Interventionists delivered insufficient numbers of review sessions due to concentration on participant recruitment. This left interventionists insufficient time for key intervention procedures. A total of 10 key changes that were made to RCT procedures are summarised.

**Conclusions:**

With improved research processes and lower monthly participant recruitment targets, a full-scale trial is feasible.

**Trial registration:**

ISRCTN13076797. Prospectively registered on 07/06/2016.

**Electronic supplementary material:**

The online version of this article (10.1186/s12890-019-0834-6) contains supplementary material, which is available to authorized users.

## Background

Cystic Fibrosis (CF) is an inherited long-term condition affecting over 80,000 people worldwide [1–5], mostly in people of Northern European ancestry [6]. People with cystic fibrosis (PWCF) typically die from lung damage at a median age of 31 years [1]. Preventative medications reduce exacerbations and preserve lung function [7–13]. There is a disparity between self-reported and objectively measured adherence to inhaled therapy, with recorded rates of 80 and 36% respectively [14]. It follows that, currently, clinicians are not able to identify people with low adherence and offer appropriate support. Low adherence predicts exacerbations requiring intravenous antibiotics (IVAB) [15, 16], which carry a risk of systemic side effects and increased mortality [17, 18], and result in higher care costs [19–21]. During 2012 the total UK spend for CF was estimated to be £110 million [22]of which £30 million was spent on inhaled antibiotics and mucolytics [23]; the following year the UK adults with CF population received 103,453 days of IVAB [24] with 54% occurring in hospital, [25].

Consistent with identified research priorities [26, 27], we developed a complex intervention to support adherence to preventive inhaled therapy. This paper summarises the intervention development process, presenting the results of the pilot randomised controlled trial (RCT) component of the feasibility study, and describing the resulting changes made to the intervention and research procedures, in advance of a full-scale RCT [28, 29].

The specific objectives of the feasibility study were:

1. To determine feasibility of a RCT based on:

- participant recruitment;
- participant retention;
- quality of primary outcome and other data at 5(+/− 1) month; and
- the acceptability and robustness of trial procedures

2. To carry out a process evaluation, consisting of quantitative and qualitative data on procedures and outcomes, in order to understand and mitigate potential sources of intervention failure in terms of contextual effects, inputs, engagement, activities and outcomes. The specifics of the process evaluation are detailed in a separate article, dedicated to this aspect of the study.
3. To document changes to research and intervention procedures for a future RCT, based on the findings. Changes to research procedures only will be documented in this report.

## Methods

### Design

The feasibility study in preparation for the full RCT consisted of a concurrent pilot RCT and a mixed methods process evaluation. The objectives of the pilot RCT were to determine feasibility of a full-scale RCT based on participant recruitment/retention, the quality of primary outcome data and the acceptability and robustness of trial procedures. This was a parallel group, open label, individually-randomised external pilot RCT with a 1:1 allocation ratio with additional semi-structured interviews. The protocol is available (Additional file 1); this report is compliant with Consolidated Standards of Reporting Trials (CONSORT) extension for randomised pilot studies [30] (Additional file 2).

### Participants

We planned to recruit 64 participants between 1st June 2016 and 30th September 2016 from two CF Centres (Nottingham University Hospitals and University Hospital Southampton). The CF registry provided a list of potentially eligible patients for each site. Medical notes were then reviewed to select those aged 16 years and over, taking – or willing to take – inhaled mucolytics or antibiotics via a nebuliser with data recording and transfer capability. We excluded those who were: post-lung transplant, on the active transplant list, receiving palliative care, lacking capacity for informed consent, or using dry powder devices to take antibiotics or mucolytics.

A purposive sample of intervention arm (*n* = 14) and control arm participants (*n* = 5), as well as interventionists (*n* = 3 0.8 WTE at each centre) and members of the wider, multi-disciplinary CF team (n = 5) was used to conduct semi-structured interviews, assessing acceptability and robustness of RCT procedures. Participants were selected based on site, age, gender and deprivation index. Postcodes were used to generate an Index of Multiple Deprivation (IMD) quintile. Service-users were selected based on objective and subjective adherence levels (respectively indicated by nebuliser-recorded inhalation data and a self-report question administered at baseline, asking “Thinking back to the last two weeks. What percentage of your nebuliser treatments have you taken?”). Professionals were selected based on site and professional category.

### Consent and randomisation

Eligible participants were invited to give written informed consent to engage in the pilot RCT. For most participants we obtained consent to have their adherence data collected beyond their active trial period, until 30 April 2017. Participants were randomised to intervention or control arms using a computer-generated pseudo-random list and random permuted blocks of varying sizes (2,4 and 6), stratified by site and number of IVAB days in the previous 12 months (≤/> 14 days) [24].

PWCF that consented to be approached for interview were contacted by letter or mail and subsequently telephone or email, dependent on preference. Professionals were contacted directly by the study team. Patients were interviewed once and interventionists were interviewed at both the beginning and the end of the study. Semi-structured interviews were conducted face-to-face, digitally audio-recorded and verbatim transcribed.

### The complex intervention

The intervention has four elements, two of which are also used for data collection purposes in the control arm of the trial

1. eTrack (PARI Pharma GmbH, Starnberg, Germany) nebulisers to deliver inhaled medications and provide monitoring functions. eTrack nebulisers send timestamped inhalation data to a 2net Hub (Qualcomm, San Diego, USA), which enables real-time monitoring. The devices could not distinguish between different drugs administered through the nebuliser; whilst it is theoretically possible for patients to press different buttons depending on the drug, our experience is that any additional step in nebuliser therapy decreases the probability that it will be completed. In addition, relying on participants pressing buttons to distinguish between drugs may have introduced an additional source of error if buttons were incorrectly used;
2. CFHealthHub server infrastructure receives the inhalation data in real-time from PARI, stores the data securely and presents this for display on the CFHealthHub apps (see Fig. 2);
3. CFHealthHub apps – with behaviour change tools and educational content to render the received data and present it to clinicians via the website, and to patients via website and mobile apps; and,
4. A manualised behaviour change intervention used by trained health professionals in their interactions with PWCF.

Both intervention and control groups received a nebuliser with data recording and transfer capability, which measured their adherence to medication. The adherence data for the control group was not visible to participants, interventionists or the care team throughout the trial. In the intervention group, the adherence data was visible to the interventionist only for a two- to four- week baseline period, following this it was also made visible to both the participants and care team. The control group continued with usual care.

The intervention group had access to: (1) information technology infrastructure which captures, stores and displays adherence data; (2) online adherence feedback and tailored modules of behaviour change content; (3) an initial visit, and at least one additional review visit, from a trained interventionist who delivered face-to-face behaviour change content. The behaviour change content was linked to online content and therapists provided support and guidance in line with, and interacting with this. Interventionists were trained through a two-day, face-to-face workshop, online learning modules and a structured four-week training programme, with an online theory test and competency assessment of the intervention delivery within the first 5 sessions.

#### Summary of the intervention development process

**Fig. 1:**
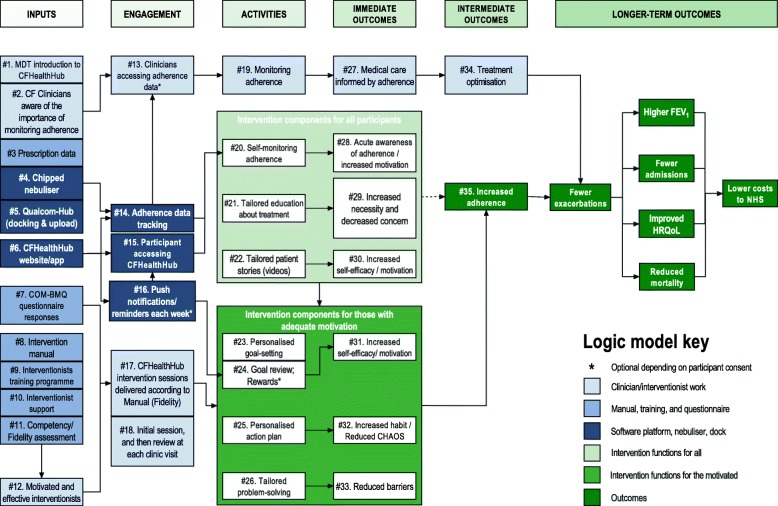
Logic model

The intervention was developed as follow: The Sheffield Microsystems Coaching Academy [31] worked with the Sheffield adult CF team to carry out a “Five Ps” strategic analysis [32]. The CF team identified their overarching purpose to be to enable PWCF to live as normal a life as possible and their objective to shift from disruptive hospital-based rescue to community-based prevention [33]. Understanding the link between medication adherence and preserved lung function [7–13], our aim became that PWCF should lose less than 2% of lung function each year. We reviewed the evidence for barriers to adherence to CF medication and the effectiveness of interventions for improving adherence [34, 35]. We used a quality improvement technique, process mapping [36], which highlighted the need for objective medication adherence data in the CF unit. Software engineers developed data capture, download and feedback systems for nebulisers. We used statistical process control to analyse data logged by nebulisers, thereby better understanding common and special cause variation in adherence [37, 38]. We modelled the use of adherence indices which take into account the percentage of the regimen taken and its appropriateness [39]. We explored barriers and beliefs about adherence [40, 41], using objective adherence data as a prompt in interviews [42] and the Theoretical Domains Framework (TDF) [43–45] to analyse the barriers and facilitators. We used the ‘COM-B’ system [46], the behaviour change wheel [47, 48] and TDF [43] to identify suitable intervention functions and behaviour change techniques with reference to two theories of behaviour: Social Cognitive Theory [49], which emphasises the importance of self-efficacy, outcome expectancies, environmental factors, and goal setting on behaviour; and, Control Theory [50], which suggests that self−/monitoring and feedback are effective in closing the gap between reality and goals, thereby promoting habit formation. We used a Markov model to estimate the incremental cost-effectiveness of adding an adherence intervention to control [51]. We produced a logic model (Fig. 1) expressing the processes by which we expected our intervention to work in terms of inputs, engagement, activities and outcomes.

**Fig. 2:**
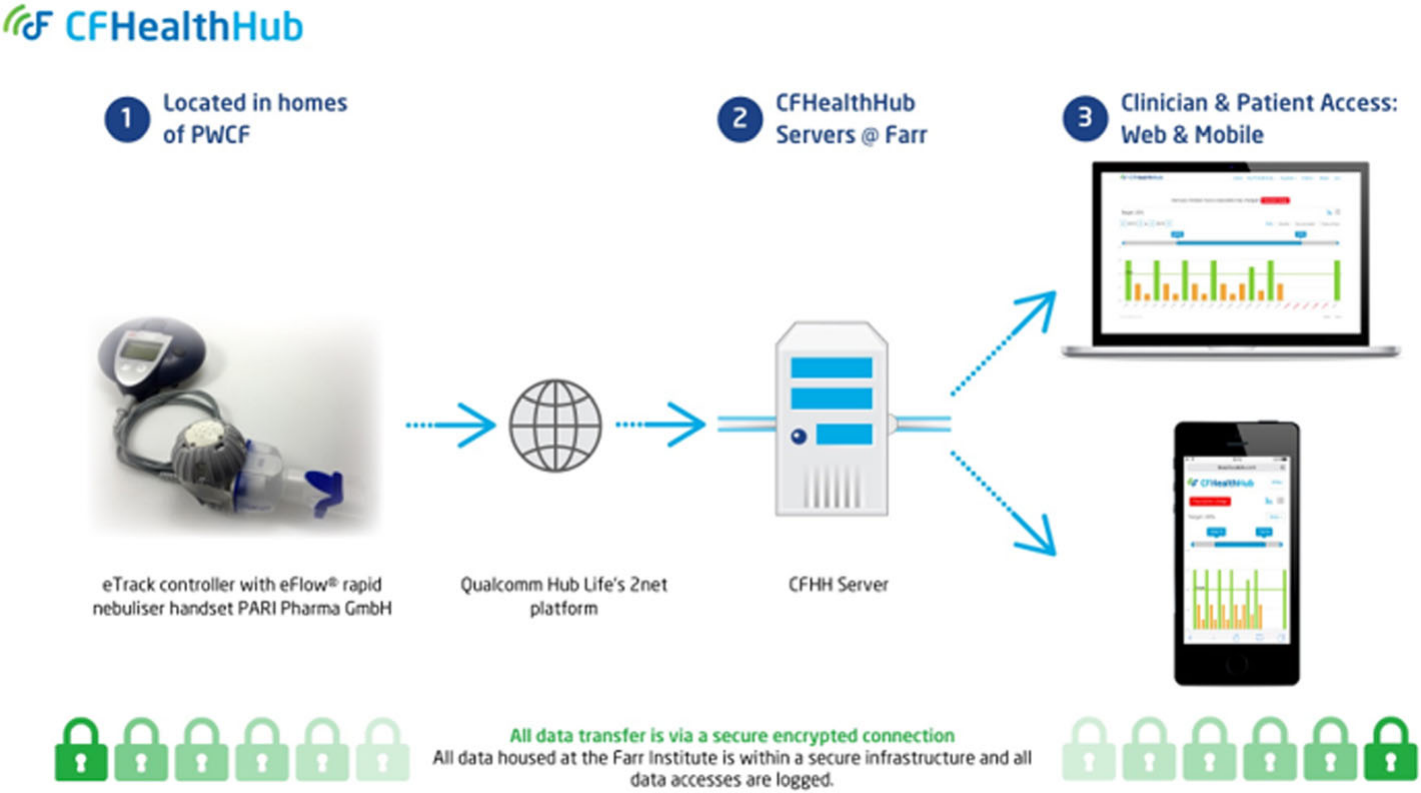
The digital platform

We produced the software platform, CFHealthHub (Fig. 2), with feedback from PWCF, clinicians, the software team and user experience (UX) design company, using Agile methods [52, 53]. The platform is compliant with regulatory, data protection, security and interoperability standards [54–57]. Following best practice guidelines [58], we used prototypes and wireframes to design the website and mobile apps [59], combining evidence-based [60] and user-centred design principles to define requirements, refine the user interface and enhance usability [61]. Short-cycle software releases allowed rapid user feedback and beta-testing of the digital platform by researchers, clinicians and patients (Additional file 3) [62–66]. Theory-based user-engagement strategies, tracking and click analytics and summaries of user ‘point-and-click’ web data, generated to describe the individual’s online activity, were built into the website and mobile apps. We incorporated short films, some which explained how medications work, others in which PWCF share experiences of forming treatment adherence habits (‘talking heads’). The digital platform has been running continuously since August 2015, receiving and presenting inhalation data in real-time from Pari eTrack nebulisers. Three physiotherapists gave verbal feedback at two time-points during the development of a training course, manual and reporting tools developed to guide and document interactions with patients using CFHH. Fidelity assessment sheets were developed for these interactions based on the National Institute for Health process [67, 68].

### Sample size

In the proposed full-scale trial, we hoped that 15 CF units would each recruit 46 patients in six months (8 patients per centre per month). To match this rate, our two-centre pilot needed to recruit 64 participants in its four-month accrual window. To progress to a full-trial our recruitment target was to consent and randomise 48 participants at two centres over four months, which was 75% of the rate required in the main trial. A second progression criterion was attrition from contribution of exacerbation data of no more than 15% of randomised participants at 5 (±1) months. This sample would also give us sufficient confidence to predict recruitment and retention in the full-scale RCT with sufficient precision [69–72].

### Outcomes

Outcomes of interest mapped directly onto the objectives:

- participant recruitment of ≥ 48 participants (75% of target) in four months;
- participant retention, defined as ≥ 85% of those randomised contributing exacerbation data at study completion;
- quality of primary outcome and other data at 5(+/− 1) month. We assessed the ability of study staff to collect valid data for the full-scale trial’s proposed primary outcome, the number of pulmonary exacerbations. Defined according to the modified Fuchs’ criteria [73], a pulmonary exacerbation was said to have occurred if a patient was treated with intravenous antibiotics for any one of 12 signs or symptoms (Additional file 1). An exacerbation form was administered by the site interventionist at each clinical encounter, with every study participant. The form captured whether there was a clinical need for a course of IVABs, which Fuchs’ criteria were met and whether participants accepted treatment IVABs. Secondary clinical outcomes are listed in Table 1 and were collected as baseline and 5(+/− 1) month post-randomisation.
- the acceptability and robustness of trial procedures, assessed through semi-structured interviews, reports recorded on our log of problems, management group and patient and public involvement (PPI) meetings.

**Table 1:** Secondary clinical outcomes

Secondary outcomes	
Body Mass Index (BMI).	
Forced expiratory volume in 1 s (FEV1): standardised spirometry as a measure of condition severity [100].	
EuroQol EQ-5D-5 L: generic health status measure for health economic analysis [101].	
The Patient Activation Measure (PAM-13): assessment of patient knowledge, skill, and confidence for self-management [102]. Elements of the PAM-13 map to logic model constructs #29 and #30.	
Confusion, Hubbub, and Order Scale (CHAOS 6-item): measure of life chaos [103]. Elements of the CHAOS-6 map to logic model construct #32.	
Medication Adherence Data-3 items (MAD-3). Bespoke questionnaire adapted from the Medication Adherence Reporting Scale [104]: 1. I forget to take my nebuliser treatment (Always/Often/Sometimes/Rarely/Never) 2. I take fewer nebuliser treatments than my doctor recommends (Always/Often/ Sometimes/Rarely/Never) 3. I decide to skip one or more of my nebuliser treatments (Always/Often/Sometimes/Rarely/Never) Maps to logic model construct #28.	
Self-Report Behavioural Automaticity Index (SRBAI) [105]. Maps to logic model construct #31.	
Cystic Fibrosis Questionnaire-Revised (CFQ-R): disease specific health-related quality of life instrument [106].	
The Patient Health Questionnaire depression scale (PHQ-8): severity measure for depressive disorders [107].	
The General Anxiety Disorder 7-item anxiety scale (GAD-7): severity measure for anxiety [108];	
The Capability Opportunity Motivation Behaviour Beliefs Questionnaire (COM- BMQ), incorporating the validated self-report Beliefs about Medicines Questionnaire - specific (Nebuliser adherence) (BMQ 21-item) [109], customised by the team to identify perceived necessities and concerns for nebuliser treatment. Elements of the COM-BMQ map to logic model constructs #7, #29 and#33.	
Project-specific items on belief, intention and confidence	
Subjective adherence single question: self-report estimate of adherence as a percentage. Maps to logic model construct #28.	
Self-reported problems: identification of capability and opportunity barriers to nebuliser adherence. Maps to logic model construct #33.	
Concomitant medications: bespoke instrument, designed for this research project.	
Resource use form (inpatient IV days; routine clinic visits; unscheduled outpatient contacts; unscheduled inpatient stays).	
Prescription: a monthly prescription check to both check for data transfer to CFHealthHub and review for an indication that the prescription has changed	
Objective Adherence to prescribed medication	
Any treatment with IV antibiotics	

There are three reasons for defining the primary outcome in terms of the modified Fuchs’ criteria [73]. First, to avoid excessive ascertainment bias in intervention group participants who would be reviewed more frequently. It follows that there is a higher chance of detecting an exacerbation in this group. By setting a sufficiently high bar, that is - an exacerbation deemed severe enough to require IVABs - we aim to prevent over-ascertainment of mild exacerbations in the intervention group. The use of IVABs is unlikely to be missed in either trial arm, since it is administered by the CF team in an acute setting, whereas oral antibiotics from general practitioner (GP) might be underreported in the control group who are seen less frequently. Second, the use of four or more symptoms - as in the original Fuchs’ criteria - is a high threshold; by comparison, our use of one or more symptoms increases sensitivity to CF symptoms. Finally, this definition is in use in modern pivotal trials [73].

### Data analysis

Details of participant screening, recruitment and retention were presented in a CONSORT flow diagram. Baseline characteristics were presented by treatment arm and overall.

The total number of exacerbations by participant and by treatment arm were presented. As this was a pilot study, definitive comparisons of interventions were not undertaken, however point estimates of effect and their 95% confidence intervals were reported for primary and secondary clinical outcomes [72, 74]. A primary effectiveness analysis was conducted on the primary clinical outcome using a negative binomial model and adjusting for number of IV days in the previous 12 months and site. Incidence rate ratio (IRR) and 95% confidence intervals were presented.

Adjusted mean difference in Forced Expiratory Volume (FEV-1) percent predicted was estimated using a multiple linear regression model adjusting for baseline and site. A full statistical methods and descriptive statistics for all secondary outcomes can be found in Additional file 4. Trial statisticians remained blind until database freeze, the point where all data had been input and all known queries resolved. The intention-to-treat population (primary analysis set) included all participants for whom consent was obtained and who were randomised to treatment, regardless of whether they received the intervention or not [75]. We tested using prescription and nebuliser data to calculate the total number of doses and simple unadjusted adherence [39]. Weekly numerator-adjusted normative adherence [39] was calculated and a mean by treatment arm was calculated and presented as a line graph. Data collected beyond the final intervention session, until 30th April enabled us to understand whether flat-lining of the adherence charts resulted from a technical fault or non-adherence to prescribed medication. The number of adverse events (AEs) and serious adverse events (SAEs) was presented by treatment arm.

Statistical analysis was performed using R version 3.4.1 statistical software [76].

A Framework analysis [77] of the semi-structured interviews was undertaken to investigate the acceptability of trial procedures for participating site staff and patients [78]; additionally, we investigated whether control participants had been “contaminated” by knowledge of, or receipt of the intervention [79].

### Approach taken to modifying research procedures

Modifications, arising from interviews, reports recorded on our log of problems, management group and patient and public involvement (PPI) meetings, were categorised to do with: the software platform; the manual and training; and, the RCT procedures. Based on a modified version of the approach taken by Bugge, we tabulated issues and solutions [29]. We regularly reviewed priorities for development of the digital platform using a system known as, “Must have, Should have, Could have, and Won’t have but would like” (MoSCoW) [28], frequently used in agile software development [52, 53].

### Patient and public involvement

The Patient and Public Involvement (PPI) Group were recruited through leaflets placed in CF units, advertising on the People in Research website and snowballing. They teleconferenced to prevent cross-infection between PWCF [80] and provided feedback on intervention data-sharing policies, usability and presentation of the website/user-guide; they piloted participant information materials and one person also provided input on the trial protocol and interview guides (Additional file 1).

### Ethical approval

The study received approval from London Brent Research Ethics Committee (16/LO/0356). The funder was not involved in the trial design, patient recruitment, data collection, analysis, interpretation, or presentation, writing or editing of the report, or the decision to submit for publication. The corresponding author had full access to all the data in the study and had final responsibility for the decision to submit for publication.

## Results

### Recruitment

**Fig. 3:**
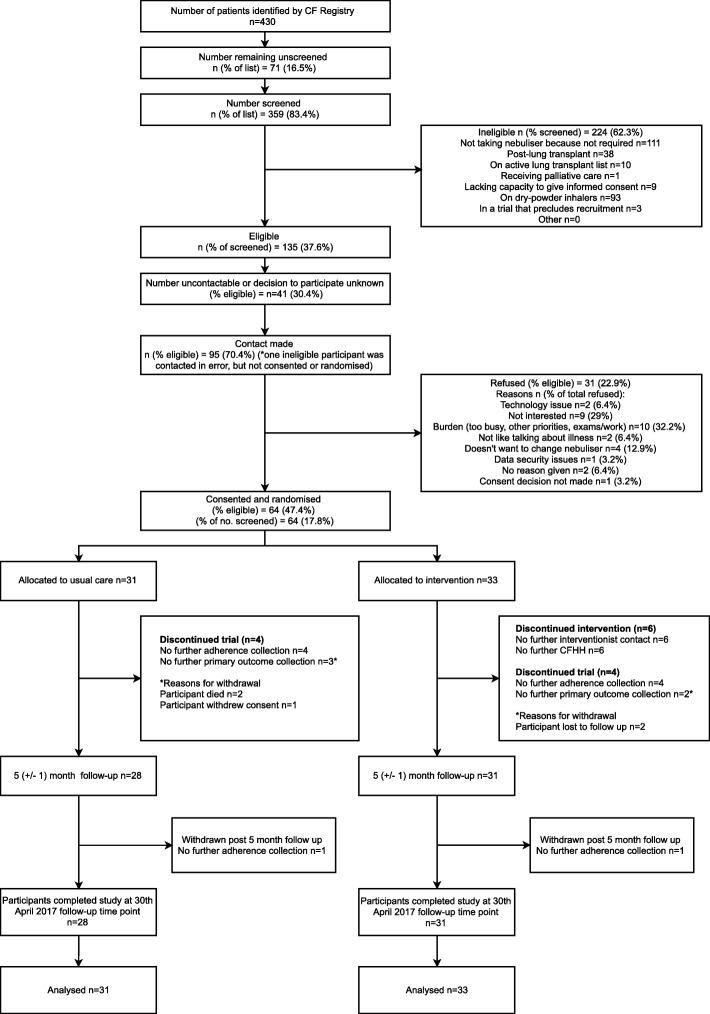
Participant flow

We recruited between 23rd June and 30 September 2016. Overall, 430 PWCF were reviewed for eligibility (Fig. 3). Of these, 135 were eligible, 95 (70%) successfully contacted and 64 (67%) of those contacted consented; 100% of the intended sample size were therefore recruited, exceeding the 75% target. A total of 33 participants were allocated to the intervention group and 31 to the control group.

#### Participant characteristics

Participants entering the study had a median age of 27 and 56% were male (Table 2). Most participants were from average (25%), low (23%) or least deprived (23%) areas of deprivation. Mean baseline FEV1 percent predicted was 57.3. Full baseline characteristics tables can be found in Additional file 4.

**Table 2:** Baseline characteristics by treatment arm

	Intervention	Control	Overall
Age			
n	33	31	64
Mean (SD)	31.6 (13.3)	27.8 (8.9)	29.7 (11.5)
Median (IQR)	28 (21,37)	26 (20,34)	27 (21,36)
Sex			
Male	18 (55%)	18 (58%)	36 (56%)
Female	15 (45%)	13 (42%)	28 (44%)
Socioeconomic Status			
Most deprived	6 (18%)	1 (3%)	7 (11%)
High deprivation	4 (12%)	7 (23%)	11 (17%)
Average	8 (24%)	8 (26%)	16 (25%)
Low deprivation	6 (18%)	9 (29%)	15 (23%)
Least deprived	9 (27%)	6 (19%)	15 (23%)
FEV1% Predicted			
n	33	31	64
Mean (SD)	53.4 (19.4)	61.4 (22.7)	57.3 (21.3)
Median (IQR)	49.2 (39.4,61.9)	53.4 (43,80)	49.6 (41.9,76.7)

### Retention

**Fig. 4:**
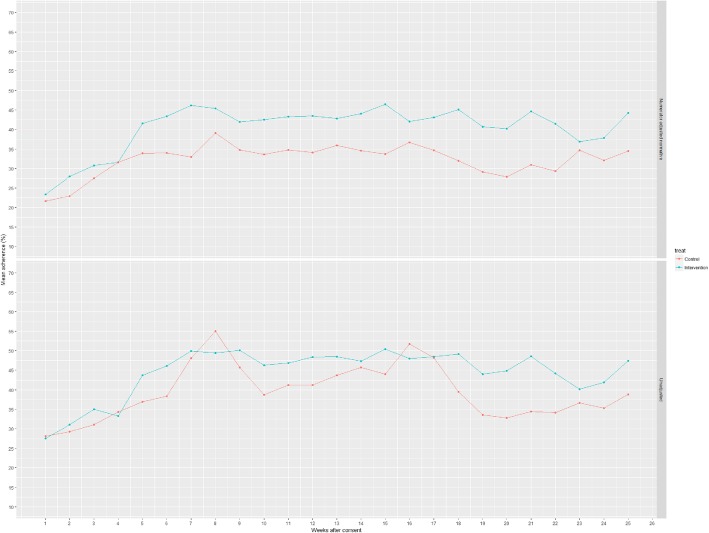
Simple unadjusted and numerator-adjusted adherence

At study completion on 30th April 2017, 60 (94%: Intervention = 32, Control =28) participants contributed exacerbation data, therefore exceeding the target 85% retention rate with respect to primary outcome data contribution. A total of 57 (89%: Intervention = 30, Control = 27) contributed FEV_1_ data; 59 (92%: Intervention = 31, Control = 28) contributed follow-up questionnaire data and 48 (75%: Intervention = 24, Control = 24) contributed 5 (±1) months of adherence data (Fig. 4). Two participants died (not related to RCT participation), one withdrew research consent, two were lost to follow-up, and two withdrew from adherence data collection (Intervention = 1, Control = 1).

### Quality of primary clinical outcome data

There were 79 data collection sheets completed providing information on, the expected primary outcome for the full trial, in those followed up for 5 (±1) months; 60 of the data collection sheets identified episodes that fulfilled the primary outcome with at least 1 Fuchs’ symptom and treatment with IV antibiotics. A total of 18 sheets were completed for episodes not treated with IVABs, and one episode treated with IVAB did not meet any of the modified Fuchs’ criteria. Of 60 that were included in the analysis set, 35 exacerbations occurred in intervention participants, 25 in control participants (see Discussion). 33 participants experienced at least 1 exacerbation (Intervention = 19 (60%), Control = 14 (50%)). Adjusted IRR was 1.12 (95% CI: 0.66–1.94) indicating no difference between treatment arms.

### Other key clinical outcomes

Adjusting for baseline and site, there was a between-group difference of 5% (95% CI -2 to 12%) in FEV_1_ percent predicted (Table 3). Figure 4 indicates a small difference across treatments arms in numerator adjusted mean weekly adherence during the study with the overall difference between treatment arms for the whole study period being 10% (95% CI: -5.2 to 25.2), greater in the intervention arm. Further details of secondary clinical outcomes are available in Additional file 4. A total of eight adverse events occurred; seven participants (11%) had at least one adverse event, of which five were serious adverse events, none related to the intervention. Of the five SAEs, 3/33 (9%) were in the intervention arm and 2/31 (7%) were in the control arm. The 2 SAEs in the control arm were deaths; there were no deaths in the intervention arm but 2 hospitalisations and 1 persistent or significant disability/incapacity.

**Table 3:** Results of clinical outcomes

		Intervention		Control			
		n	Median (IQR)	n	Median (IQR)	IRR	95% CI
Number of exacerbations treated with IV antibiotics with at least 1 Fuchs’ criteria in a 6 month period (primary outcome definition)	Unadjusted	32	1 (0, 2)	28	0.5 (0, 2)	1.22	(0.69,2.21)
	Adjusted^a^					1.12	(0.66,1.94)
		n	Mean (SD)	n	Mean (SD)	Mean Diff	95% CI
FEV1 percent predicted	Unadjusted	30	54.2 (21.1)	27	59 (23.9)	−4.8	(−17,7.1)
	Adjusted^b^					5	(−2,12)

^a^Adjusted for number of IV days in previous 12 months and site

^b^Adjusted for baseline and site

### Acceptability and robustness of the trial procedures

The information sheet was described as ‘wordy’ and questionnaires repetitive; otherwise, participants found research procedures acceptable. Interventionists confirmed that they had no access to adherence data for control patients, as required by trial procedures. MDT members said a lack of resources and training precluded contamination. There was no systematic strategy to involve the wider CF team in the use of the adherence data to support care and most data sharing was via the interventionist. This was partly a conscious strategy to avoid contamination of the control subjects. Aside from the new nebuliser and 2net Hub, control participants viewed their care and approach to self-management as unaffected, although one admitted making an effort to adhere because of the collection of adherence data.

### Modifications to research procedures

Additional file 5 documents 10 technical changes made, in relation to RCT procedures, to CFHH (*n* = 1), IT infrastructure (*n* = 1), and trial procedures (*n* = 8). To prevent adherence data flatlines, nebulisers **(#4)** and 2net Hubs **(#5)** are now paired at the factory. In the feasibility study, a focus on RCT recruitment targets gave interventionists inadequate time to deliver review visits **(#18, #24)**, critical for updating personalised action plans **(#25)** and updating coping plans **(#26)**. Therefore the full-scale trial will also have a longer pro rata participant accrual window. As there was inconsistency in completion of the case report form for exacerbations (primary clinical outcome), changes have been made to training and the case report forms for the main trial. Namely, the modified form records if the clinical team considered there to be a need for IVABs, whether these were accepted or not and subsequently whether any Fuchs’ criteria were met. Fuchs’ criteria are assessed regardless of whether IVABs were accepted by the patient.

## Discussion

The pilot RCT recruited well; in particular, recruitment to the pilot was very high compared to other clinical CF trials, illustrating the importance and relevance of adherence to CF patients [81]. Participant retention and study procedures were satisfactory, with no obvious contamination in the control arm.

In 24% of cases where clinicians competed the case report form for an exacerbation, the criteria for this primary clinical endpoint were not met (see Results | Quality of primary clinical outcome data). This may have been, as in other studies [82, 83], instances in which a clinician identified the clinical need for IVAB but patients chose treatment with oral antibiotics. There is no universally-agreed definition of an exacerbation [84, 85]. In our study, the modified Fuchs’ criteria provided a relatively objective assessment of clinical need; adding treatment with IVABs ensured that only clinically severe episodes are analysed. This definition goes some way to decreasing ascertainment bias arising when closer monitoring identifies less serious cases [84, 85], with less impact on the health system than IVAB, and allows adjustment for baseline adherence [86] via routinely collected data. However, as adherence improves, patients may be more willing to accept IVABs for exacerbations, which could make ascertainment bias more marked. There was no difference in the rate of exacerbations/primary outcome between treatment arms however the study was not powered to detect an effect and was designed to assess feasibility and the quality of the primary outcome data as described above. Changes to CRF exacerbation forms for the full-scale RCT will capture the number of cases in which exacerbation criteria were met but IVABs were refused by the patient, allowing detection of any ascertainment bias. To better understand variation in adherence over time, the full-scale trial will use differences in mean group adherence over time, as expressed in Fig. 4, in addition to pre/post measurements, which can miss changes in adherence over the course of the trial **(#35)**. Future intervention research should consider the use of continuous data to avoid missing patterns of adherence behaviour between assessment timepoints.

Evidence that patient access to data improves health outcomes or is cost-effective is generally poor and lacks information about context and implementation [87, 88]. The full-scale RCT of our modified intervention at 19 UK centres (ISRCTN55504164) will provide high quality evidence on the subject in 2020, with further process evaluation and health-economic modelling. The problem of how to embed routine use of adherence data by healthcare professionals [89–93] is the subject of the CFHealthHub Data Observatory (ISRCTN14464661). This quality improvement project, onto which pilot sites have now transitioned, will eventually host sites from the full-scale RCT. The data it collects will be used to develop theory and practical guidance about the collaborative use of adherence data that is generalisable to multiple contexts [94, 95], with the aim of improving patient care and using NHS resources more efficiently [96]. The Observatory will act as a platform for efficient trials [97, 98]. Shared processes and improvement activities should increase the capacity and success of participating cystic fibrosis clinical research teams [99].

## Conclusions

We have developed a theory-based complex intervention to help PWCF adhere to their medication. The pilot trial observed a recruitment rate of eight participants per centre per month, with acceptable levels of attrition. The findings reported in this paper give high levels of confidence that, with improved trial procedures, a full-scale trial is feasible.

## Additional files

Additional file 1: Study protocol and interview guides. (PDF 2311 kb)

Additional file 2: CONSORT checklist. (DOC 226 kb)

Additional file 3: Release notes for 59 software development cycles between June 2015 and June 2017. (DOCX 17 kb)

Additional file 4: Statistical methods, outcomes and estimation. (DOCX 272 kb)

Additional file 5: Changes to intervention and trial procedures. (DOCX 22 kb)
