# Supplementary material for: Supporting medication adherence for adults with cystic fibrosis: a randomised feasibility study
Source: BMC Pulm Med. 2019 Apr 11;19:77. doi: 10.1186/s12890-019-0834-6 (PMC6458785; doi:10.1186/s12890-019-0834-6)
Supplement: Supplementary file 1 — Study protocol and interview guides. (PDF 2311 kb) [file 12890_2019_834_MOESM1_ESM.pdf]

ACtiF

Development and evaluation of an intervention to support Adherence to treatment in adults with Cystic Fibrosis

A feasibility study comprised of an external pilot randomised controlled trial and process evaluation

RESEARCH PROTOCOL
V3.1 16Nov16

**Sheffield CTRU**	J13-002
**IRAS**	199775
**ISRCTN**	ISRCTN13076797
**Authorised by:**	Martin Wildman
	Alicia O'Cathain

Funding

This project was funded by the Programme Grants for Applied Research programme (RP-PG-1212-20015) and will be published in full in the NIHR Journals Library. This research presents independent research commissioned by the NIHR. The views and opinions expressed by authors in this publication are those of the authors and do not necessarily reflect those of the NHS, the NIHR, MRC, CCF, NETSCC, the Health Technology Assessment programme or the Department of Health.

Roles and responsibilities

Names, affiliations, and roles of protocol contributors

The following all contributed to drafting elements throughout the protocol: Martin Wildman, Consultant Respiratory Medic; Marlene Hutchings, Physiotherapist (Sheffield Teaching Hospitals NHS Foundation Trust); Madelynne Arden, Professor of Health Psychology (Sheffield Hallam University); Alicia O'Cathain, Professor of Health Services Research; Sarah Drabble, Research Associate, Stephen Walters (Professor of Medical Statistics and Clinical Trials); Paul Tappenden, Reader in Health Economics; Daniel Beever, Research Associate and Patient Representative; Pauline Wheelan mHealth Applications Manager (University of Manchester); Judy Bradley, Professor of Physiotherapy and Health Services Research; Hannah Cantrill (Research Associate); Chin Maguire (Study Manager); and, Daniel Hind, Assistant Director, Sheffield Clinical Trials Research Unit (University of Sheffield). MW and MA are guarantors of the intervention description; SW is the guarantor of the statistical design and analysis plan; DH is the guarantor of other elements of the pilot trial; AO'C is guarantor for the qualitative research components.

Sponsor

Sheffield Teaching Hospitals NHS Foundation Trust
D Floor
Clinical Research Office
Royal Hallamshire Hospital
Glossop Road
Sheffield
S10 2JF
Tel: (0114) 2256424
Fax: (0114)

Role of study sponsor and funders

Neither the funder nor the sponsor have had any role in study design, data collection and analysis, decision to publish, or preparation of manuscripts.

Key Contacts

Study Manager

Chin Maguire
Clinical Trials Research Unit
The University of Sheffield
School of Health and Related Research
30 Regent Street
Sheffield
S1 4DA
Tel: (0114) 222 0717
Fax: (0114) 222 0870
e-mail: c.maguire@sheffield.ac.uk

Chief Investigator

Dr Martin Wildman
Adult CF Centre
Northern General Hospital
Herries Road
Sheffield
S5 7AU

Tel: (0114) 2715212
Fax: (0114) 222 0870
e-mail: Martin.Wildman@sth.nhs.uk

Composition, roles, and responsibilities

Project Management Group

Name	Role	Institution
Dr Martin Wildman	Chief Investigator	Sheffield Teaching Hospitals NHS Foundation Trust
Professor Alicia O'Cathain	Chief Investigator	University of Sheffield
Dr Daniel Hind	CTRU Assistant Director	University of Sheffield
Chin Maguire	Study Manager	University of Sheffield
Hannah Cantrill	Research Assistant	University of Sheffield
Helen Wakefield	Trial Support Office	University of Sheffield
Dan Beever	PPI Lead	University of Sheffield
Dr Sarah Drabble	Qualitative Researcher	University of Sheffield
Professor Maddy Arden	Professor of Health Psychology	Sheffield Hallam University
Marlene Hutchings	Research Physiotherapist	Sheffield Teaching Hospitals NHS Foundation Trust
Professor Judy Bradley	Professor in Physiotherapy	Queens University Belfast

Independent Project Steering Committee

Name	Role	Institution
Professor Kathy Rowan	Independent Chair	London School of Hygiene and Tropical Medicine
Professor Sarah Garner	Independent member	The National Institute for Health and Care Excellence
Professor David Torgerson	Independent Member	University of York
Dr Lisa Hampson	Independent Statistician	Lancaster University
Dominic Kavanagh	PPI Representative	
Dr Martin Wildman	Chief Investigator	Sheffield Teaching Hospitals NHS Foundation Trust
Professor Alicia O'Cathain	Chief Investigator	University of Sheffield
Dr Daniel Hind	CTRU Assistant Director	University of Sheffield
Chin Maguire	Study Manager	University of Sheffield
Professor Maddy Arden	Professor of Health Psychology	Sheffield Hallam University

Contents

Funding 2
Names, affiliations, and roles of protocol contributors 2
Sponsor 2
Role of study sponsor and funders..2
Composition, roles, and responsibilities 3
Project Management Group 3
Independent Project Steering Committee 4
Contents 5
Figures 7
Tables..7
1. Lay summary 8
2. Introduction..9
2.1 Background...9
2.2 Rationale 9
3. Aim and objectives 11
3.1 Aims..11
3.2 Objectives 11
4. Design 12
5. Participants and study settings...13
5.1 Settings and locations where the data will be collected.....................................13
5.2 Eligibility 13
5.2.1 Inclusion criteria for participants...13
5.2.2 Exclusion criteria for participants..13
5.2.3 Eligibility criteria for study centres 13
5.2.4 Eligibility criteria for interventionists..13
6. Interventions 14
6.1 Summary...14
6.2 Microchipped devices 15
6.2.1 The eTrack nebuliser system (Pari GmbH) 15
6.2.2 The Bi-neb AAD System from (Philips Healthcare) 15
6.3 Information technology infrastructure 15
6.3.1 The Qualcomm hub 15
6.3.2 CFHealthHub..15
6.3.3 The Bi-Neb data transfer system 16
6.4 The Behaviour Change Intervention (BCI) 17
6.4.1 Rationale and theory 17
6.4.2 Intervention providers...22
6.4.3 Materials 22
6.4.4 Procedures...22
6.5 Usual care 27
6.6 Criteria for discontinuing or modifying allocated interventions 27
6.7 Strategies to improve adherence to intervention protocols..................................28
6.7.1 For health professionals...28
6.7.2 For patients 28
6.8 Relevant permitted / prohibited concomitant care...28
7. Outcomes 29
7.1 Feasibility outcomes (‘stop-go’ or ‘success’ criteria for RCT) 29
7.2 Process data relating to the implementation of the trial.......................................29

7.3 Process data relating to the implementation of the intervention....................................30
7.4 Clinical outcomes and covariates 31
7.4.1 Primary clinical outcome 31
7.4.2 Secondary clinical outcomes 32
7.4.3 Adherence to prescribed medication..33
8. Sampling 37
8.1 Quantitative components 37
8.1.1 Sites..37
8.1.2 Sample size 37
8.1.3 Approach, non-participation and recruitment..37
8.2 Qualitative components 38
9. Assignment of interventions 38
9.1 Sequence generation 38
9.2 Allocation concealment 38
9.3 Implementation 38
9.4 Blinding 38
10. Data collection, management and analysis 39
10.1 Quantitative data 39
10.1.1 Data collection methods..39
10.1.2 Data Management 40
10.1.3 Data quality assurance 40
10.2 Qualitative data 41
10.2.1 Audio recordings of consultations 41
10.2.2 Semi-structured interviews: participants 41
10.2.3 Semi-structured interviews: professionals 41
10.2.4 Undertaking the interviews 41
11. Data analysis 42
11.1 Quantitative analysis...42
11.2 Qualitative analysis..43
11.3 Combining data and findings from the different components 44
12. Monitoring 44
12.1 Oversight..44
12.2 Description of any interim analyses and stopping guidelines.......................................45
12.3 Harms (safety assessments) 45
12.3.1 Serious Adverse Events 45
12.3.2 Adverse events we require reporting: 46
12.3.3 Expected SAEs and adverse events 46
12.3.4 Reporting 47
12.4 Auditing 47
12.5 Finance and indemnity...47
13. Ethics and dissemination 48
13.1 Approvals...48
13.2 Protocol amendments...48
13.3 Consent 48
13.4 Confidentiality 49
13.5 Declaration of Interests..49
13.6 Access to data 49
13.7 Ancilliary and post-trial care 49
13.8 Dissemination policy 49
References..51
Appendix 1. W.H.O. Trial Registration Data Set 56

Figures

Figure 1. Interaction between complex intervention components 14
Figure 2. Interactions between capability, opportunity and motivation 17
Figure 3. Habit formation incorporating COM-B and necessities and concerns 18
Figure 4. Interplay between COM-B components during habit formation....18
Figure 5. Behaviour change intervention flow chart 26
Figure 6. Participant timeline for the external pilot RCT 36
Figure 7. Assumptions of the MRC Guidance on Process Evaluation 43

Tables

Table 1. Learning objectives of the CFHealthHub modules 16
Table 2: Intervention modules 20
Table 3. Individual-level data derived from PWCF and sites....34
Table 4. CFHealthHub data (research arm only) 35

1. Lay summary

Cystic Fibrosis (CF) is an inherited disease affecting 10000 people in the UK with an average age at death of 28 years in 2012. The lungs of people with CF (PWCF) are prone to infections. Daily physiotherapy and inhaled medications are needed to stay healthy. Around £30 million is spent annually on inhaled therapy but average adherence has been shown to be only 36%. Data suggest that adherence is better in younger children (71% in under 12s, falling to 50% in teenagers) but of the 10000 UK PWCF almost 6000 are now adults. PWCF who collect <50% of their medication cost the healthcare system significantly more than PWCF who collect more than 80% and most of the additional cost results from unscheduled emergency care and hospital admission. This unscheduled emergency care is distressing for PWCF and their families.

We have designed an intervention to help adult PWCF see how much treatment they use. We use dose-counting nebulisers to collect data and send it to a website where it can be displayed. We have worked with PWCF to make the information easy to understand. The website has modules which teach PWCF how to build successful treatment habits. We have developed a toolkit to help PWCF and a health professional (interventionist) work together to form habits of adherence to treatment.

The NHS should not fund this intervention without its effectiveness and value for money being evaluated in a Randomised Controlled Trial (RCT). However, there is currently insufficient information to effectively plan or justify funding a RCT on the scale required. This feasibility study is an essential preliminary to the full scale RCT. The purpose of this feasibility study is to see whether the proposed procedures for the full scale RCT are feasible and acceptable to PWCF. It will also tell us whether the intervention can be delivered by health professionals and is acceptable to PWCF, outside the NHS trust where it was developed.

We will recruit PWCF for four months at two CF units. We hope we will recruit 64 PWCF overall, but will deem the full scale RCT feasible if we recruit 48. A computer will decide whether people who consent to be in the study will receive usual care alone or also receive the intervention. Both groups have a short period of two to four weeks when data is collected through their nebulisers and fed back to the website. It is only after that period that those allocated to the intervention are allowed to use the website and receive enhanced care from the interventionist. After that point, all participants are followed up for 5 (+/-) months. Participants will complete a series of questionnaires at the outset and at 5 (+/-) months.

With appropriate consent, the interventionist or member of the multidisciplinary team (MDT) will audio record consultations between themselves and PWCF who are receiving the intervention or usual care. Qualitative researchers will conduct: 20-24 interviews with PWCF receiving the intervention; 20-24 interviews with PWCF receiving usual care; eight interviews with the four health professionals who are delivering the intervention; and eight semi-structured interviews with members of the wider MDT. These interviews are intended to help the team understand and mitigate potential sources of failure in the intervention and the proposed full-scale trial.

2. Introduction

2.1 Background

Cystic Fibrosis (CF) is a long term condition (LTC) in which poor adherence to high cost drugs shortens lives and increases NHS costs. CF is a LTC affecting 10,000 people in the UK with PWCF typically dying from lung damage at a median age of 28 years [1]. Randomised controlled trials show that preventative medications reduce exacerbations and/or preserve lung function, [2–8] however adherence is poor. A recent review of objective measures of adherence using medicine possession ratios (MPR: prescriptions collected over prescriptions issued) and instrumented medication monitors showed adherence ranging from 67% for oral antibiotics, 31-53% for inhaled antibiotics, 53-79% for mucolytics agents and 41-72% for hypertonic saline [9]. Accumulating evidence suggests poor adherence is associated with poor outcomes. PWCF collecting four or more courses of alternate month nebulised tobramycin per year were 60% less likely to be admitted to hospital than PWCF collecting one or less [10]. Lower composite MPR predicted exacerbations requiring intravenous antibiotics (IVAB) [9] and over a 12 month period PWCF with an MPR of 80% had significantly lower total healthcare costs than PWCF with an MPR <50% with a cost difference $14,211 per patient and most excess costs related to hospital care [11] . Rescue therapy with IVAB can cause renal failure [12]. The total 2012 UK spend for CF was estimated to be £100 million of which £30 million was spent on inhaled antibiotics and mucolytics [13]. Although patient self-reported adherence to inhaled therapy was 80%, objective measurement showed median adherence was only 36% and the clinicians were unable to predict which PWCF were able to successfully adhere [14] making adherence support difficult. In 2012, the UK CF population received 171,907 days of IVAB with the 93,455 of these that occurred in hospital costing an estimated £27 million [15] . It is recommended that adherence interventions should be targeted where adherence really matters [16] and targeting support towards the high cost inhaled preventative drugs in CF (median adherence 36%) has the potential to impact on the 171,907 days of IVAB a proportion of which will represent rescue therapy necessitated by failed prevention.

2.2 Rationale

The National Institute for Health Research have commissioned a Programme Grant for Applied Research to systematically develop and evaluate an adherence intervention for PWCF. The Programme Grant has three work packages

Work package 1: Build IT infrastructure to capture adherence data from nebulisers. Co-produce a web-portal, ‘CFHealthHub’, with PWCF and clinicians, in order to display routinely collected adherence data for the use of both groups.

Work package 2: Develop a toolkit based on psychological theory that can support PWCF to adhere to treatment. This will include feedback of measured adherence data and personalised interventions to increase adherence delivered through CFHealthHub. Manualise a Behaviour Change Intervention (BCI) for use by health professionals and PWCF.
All four work packages have received a favourable opinion from an NHS REC:

- Work package 2.1A: A study of the views of people with cystic fibrosis about their condition and treatments (Hampshire A REC: 14/SC/1455; IRAS: 171049);

- Work package 2.1C: A study to produce videos for the CFHealthHub website (Camden & Kings Cross REC: 15/LO/0944; IRAS: 182367);

- Work package 2.2B: A study to develop a Behaviour Change Intervention (BCI) to help patients with CF manage treatment adherence ((South Yorkshire REC: 15/YH/0332; IRAS: 184477); and,

- Work package 2.2B(1): A study to understand how to use the eTrack and Bi-neb nebuliser to help people with CF to manage their inhalation treatments (West of Scotland REC 5: 15/WS/0089; IRAS: 177900).

Work package 3: Evaluate the toolkit developed in work package 2. The planned definitive evaluation will take place in a large-scale, multi-centre Randomised Controlled Trial (RCT). The definitive evaluation will compare usual care plus staff training in the importance of knowledge, skills and confidence building for adherence versus the same plus the structured behaviour change in intervention (CFHealthHub plus manual).

There is too little information available to effectively plan or justify funding a full scale RCT. We wish to conduct feasibility study comprising of:

- an ‘external pilot RCT’ to establish the feasibility of recruitment to a larger, definitive study; and,

- a ‘process evaluation’ which will help us understand the strengths and weaknesses of both the intervention and research protocols, and ways of addressing any weaknesses.

3. Aim and objectives

3.1 Aims

The principal aims of this feasibility study are to assess the feasibility and acceptability of:

- a complex intervention, when delivered outside the team which conceived and developed it; and,
- procedures for a full-scale RCT.

3.2 Objectives

1. An external pilot randomised controlled trial to determine feasibility of a randomised controlled trial based on objective stop-go criteria (Section 7.1) related to:

(a) participant recruitment;

(b) participant retention; and,

(c) quality of primary outcome data at 5(+/- 1) month.

2. A process evaluation, relating quantitative and qualitative data on procedures to outcomes, in order to understand and mitigate potential sources of failure in:

(a) the intervention; and,

(b) the full trial.

4. Design

Mixed-methods study comprising of:

- Quantitative component: parallel group, open labelled, external pilot RCT;

  and,

- Qualitative component: analysis of audio-recorded consultations and interviews.

Quantitative and qualitative data will contribute to the process evaluation.

5. Participants and study settings

5.1 Settings and locations where the data will be collected

Nebuliser adherence data and information derived from CFHealthHub will be automatically uploaded by participants nebulisers in their own home. Data collection involving patient notes and patient reported outcome measures will take place in two specialist CF units which have not been involved in the development of the intervention. Exacerbation data will be collected by the ACtiF trial interventionist and clinicians at sites from participant notes.

5.2 Eligibility

5.2.1 Inclusion criteria for participants

1. Diagnosed with CF and with data within the CF registry
2. Aged 16 years and above
3. Taking inhaled mucolytics or antibiotics via a chipped nebuliser (e.g. eTrack or Bi-Neb) or able and willing to take via eTrack or Bi-Neb.

5.2.2 Exclusion criteria for participants

1. Post-lung transplant
2. People on the active lung transplant list
3. Patients receiving palliative care, Lacking in capacity to give informed consent
4. Using dry powder devices to take antibiotics or mucolytics

5.2.3 Eligibility criteria for study centres

1. Adult CF Centre;
2. Recognised by commissioners
3. Receiving year-of-care funding

5.2.4 Eligibility criteria for interventionists

1. Health care professional e.g. registered nurse, physiotherapist or other appropriately skilled individual such as a psychology graduate able to work at NHS Agenda for Change Band - 4 or above

6. Interventions

6.1 Summary

In the external pilot RCT, we will test procedures for a full trial. This involves allocation of PWCF to either a complex intervention or usual care. A ‘complex intervention’ is defined as one with several interacting components [17]. The complex intervention under evaluation has three broad categories of components (Figure 1):

(a) ***a microchipped device*** (nebuliser) for delivering inhaled medications, which are routinely prescribed for the control of cystic fibrosis (Section 6.2);

(b) ***information technology infrastructure*** to capture and store adherence data from the nebulisers and display it to PWCF and the CF team (Section 6.3); and,

(c) ***the behaviour change intervention***, comprising a software platform (‘CFHealthHub’ mobile apps and website) offering adherence feedback and tailored modules of content and tools used by the health professional in interactions with PWCF (Section 6.4) and accessed independently by PWCF via CFHealthHub

Services received as usual care described in Section 6.5.

Figure 1. Interaction between complex intervention components

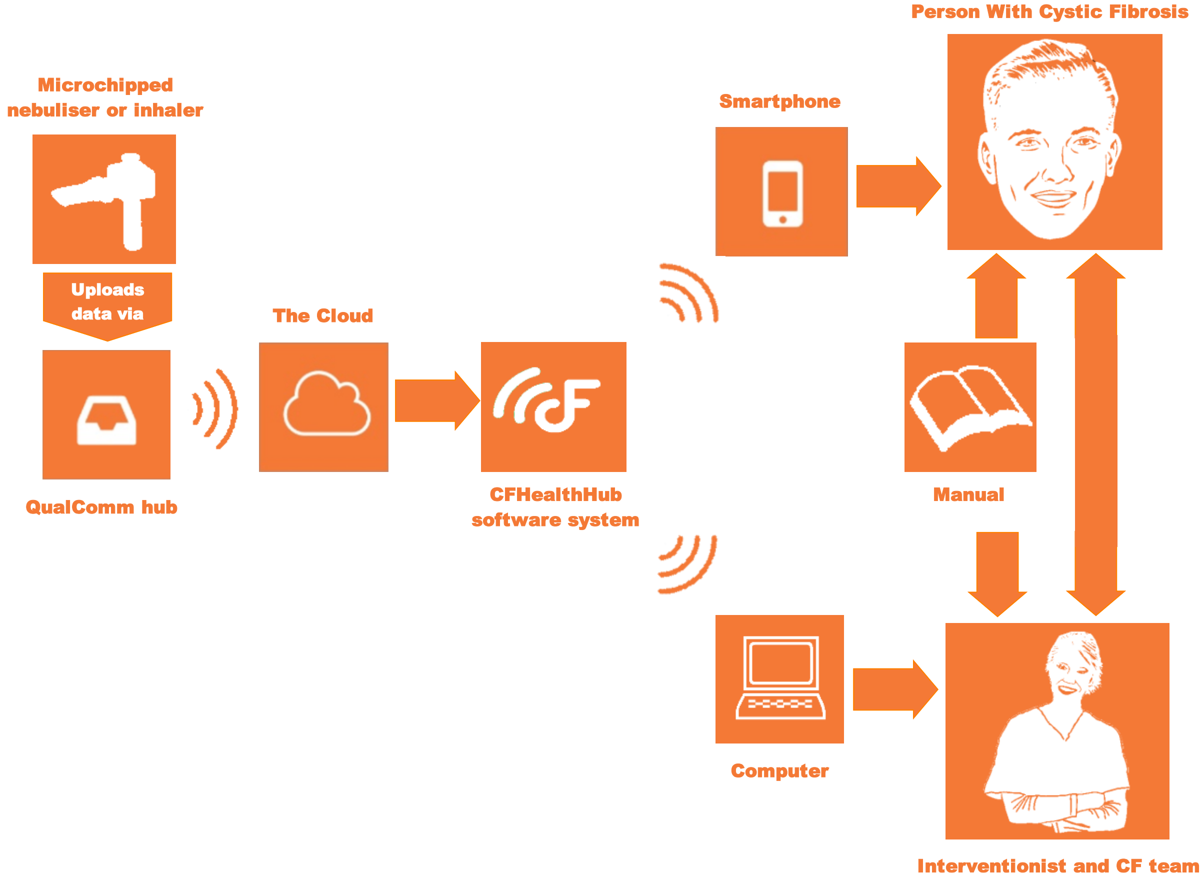

6.2 Microchipped devices

Depending on treatment strategies at different centres the participant may use an eTrack nebuliser system (Section 6.2.1), an Bi-neb AAD System from (Section 6.2.2).

6.2.1 The eTrack nebuliser system (Pari GmbH)

The eTrack controller is a modified version of the eBase controller and can be used to operate both the eFlow rapid nebulizer or Altera nebulizer. Compared to the eBase controller the eTrack is equipped with a Bluetooth chip and has a monitoring function to allow the capture of inhalation adherence data. The eFlow rapid nebuliser with eTrack controller is a CE marked medical device to be used for inhalation therapy. The device allows medications (approved for inhalation) to be transported deep into the lungs.

6.2.2 The Bi-neb AAD System from (Philips Healthcare)

The Bi-neb AAD system is a CE marked medical device which is intended for use to deliver aerosolised liquid medications for participants with cystic fibrosis. The drug delivery device is small and battery powered designed to deliver a precise dose of drug into patient's lungs. The Bi-neb AAD system is designed to deliver liquid medications that are specifically approved for use with the Bi-neb AAD System.

6.3 Information technology infrastructure

The information technology infrastructure for the complex intervention comprises:

i. The Qualcomm hub (Section 6.3.1)
ii. CFHealthHub (Section 6.3.2).
iii. The Bi-Neb data transfer system (6.3.3)

6.3.1 The Qualcomm hub

The Qualcomm hub (Qualcomm; Cambridge, UK) is a wireless device which acquires data from the chipped device and transmits it to a cloud-based data centre. It is a Class I MDD and CE registered in Europe. It is designed, developed and manufactured in accordance with a quality system compliant with ISO13485 standards, meaning it aligns with the quality requirements of international regulatory agencies in the health care industry.

6.3.2 CFHealthHub

CFHealthHub is a web-portal which displays adherence data and provides resources and tools to people with cystic fibrosis and health professionals in order to support improved nebuliser adherence. It is available on-line via computers, tablets or mobile phones.

A qualitative study (WP 2.1A) to identify the barriers and facilitators of nebuliser use in PWCF informed the development of an intervention designed to increase nebuliser adherence. Analysis of the interview data was conducted using the COM-B framework, and these findings were used to inform the development of a complex intervention centred around the feedback of objective adherence data. The intervention was further developed and refined in consultation with PWCF and clinicians. An iterative study in which prototype versions of the intervention were delivered to and reviewed by PWCF was conducted. In that iterative study we interviewed PWCF and interventionists about the usability and tailoring of the

intervention, and made improvements to the process and materials based on this feedback. The system has been developed to ensure it meets the requirements of the Data Protection Act 1998. It is intended that data on maintenance and relapse will be generated during the full scale trial.

CFHealthHub has a number of modules addressing barriers to adherence based on the COM-B system described in greater detail in Section 6.4.1. The objectives of the modules as mapped to the COM-B are outlined in Table 1 below.

Table 1. Learning objectives of the CFHealthHub modules

COM-B model component	Objectives
Physical capability	- Have the skills to be able to use the nebuliser correctly
Psychological capability	- Understand the importance of nebuliser use in CF treatment<br>- Be able to remember to use nebuliser<br>- Be able to self-monitor nebuliser use<br>- Be aware of a need to improve nebuliser use
Physical opportunity	- Have a realistic medication plan<br>- Have a working/functioning nebuliser<br>- Have a suitable place to use nebuliser<br>- Have the time to use nebuliser
Social opportunity	- Be/feel supported by others to use nebuliser
Reflective motivation	- Perceive benefits of nebuliser use<br>- Perceive few/no concerns about nebuliser use<br>- Understand the health consequences of use/non-use<br>- Feel confident about nebuliser use<br>- Intend to use nebuliser
Automatic motivation	- Have an established routine for nebuliser use<br>- Have a habit to use nebuliser

6.3.3 The Bi-Neb data transfer system

The Bi-Neb Bluetooth data transfer system is intended to automatically extract breathing device use (adherence data) from the device (Bi-Neb) via a Smartphone hub and a secure data server onto CFHealthHub. Providing the Bi-Neb is within the Bluetooth range within the patient's house, the system can retrieve this data once a day.

6.4 The Behaviour Change Intervention (BCI)

6.4.1 Rationale and theory

The rationale of the BCI is to help CF patients to self-manage their condition and to form habits that will improve adherence to their medication, thereby extending life and improving quality of life. The MRC framework for developing and evaluating complex interventions recommends that intervention development should be informed by a suitable theoretical framework and evidence base [17]. The theoretical model adopted is the COM-B model [18] which describes a 'behaviour system' of the essential and interacting conditions of Capability, Opportunity, and Motivation [18] . The model posits that non-adherence is either non-intentional (a problem of capability or opportunity or intentional (a problem of motivation). The model has been adapted to nebuliser adherence on the basis of evidence about the factors influencing nebuliser adherence in PWCF [19–32], input from expert clinicians currently delivering services to PWCF, as well as from the PPI panel and exploratory research conducted in Sheffield. It is important that interventions are tailored to individual needs and use a multi-modal approach [33]. Each of the conditions of Capability, Opportunity and Motivation has been considered in turn in the development of our intervention. The primary component of the intervention is adherence feedback delivered via the CFHealthHub. Evidence suggests that while personalised feedback can have an effect size of up to 20% in increasing adherence [34, 35], feedback is most effective when combined with additional behaviour change techniques [34].

Figure 2. Interactions between capability, opportunity and motivation

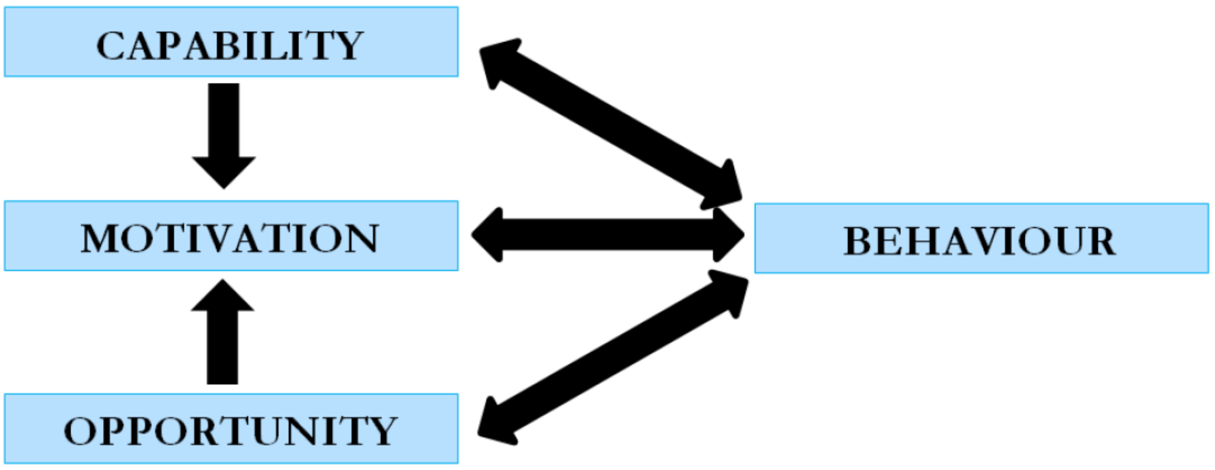

Figure 3. Habit formation incorporating COM-B and necessities and concerns

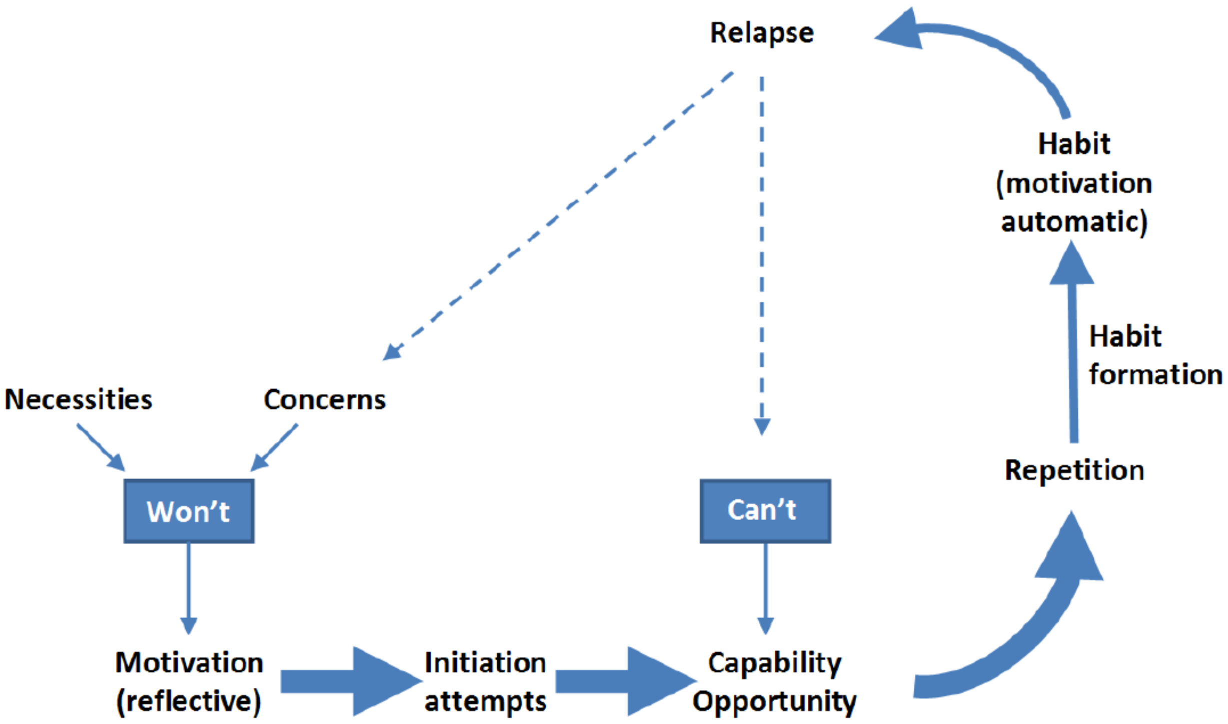

Figure 4. Interplay between COM-B components during habit formation

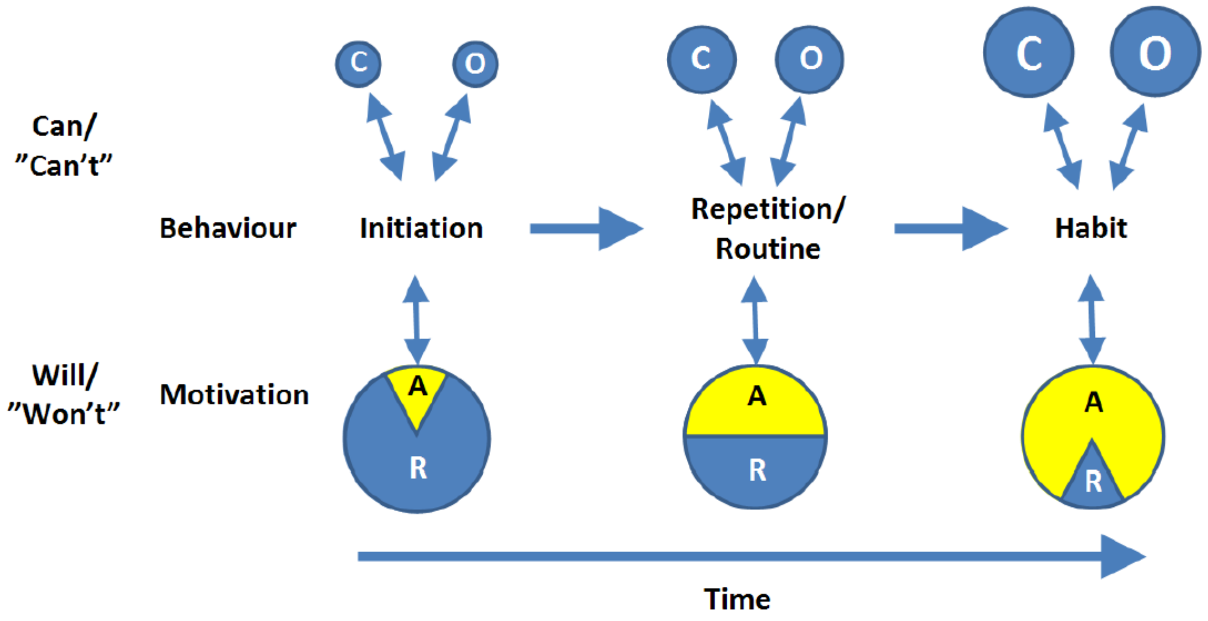

A= Automatic, R= Reflective, C= Capability, O=Opportunity

The identification and choice of appropriate behaviour change techniques has been driven by the Behaviour Change Wheel framework for the development of interventions [Michie, S. F., Atkins, L., & West, R. (2015). The behaviour change wheel: a guide to designing interventions.] which outlines a process of intervention design using the COM-B model "through the systematic evaluation of theory and evidence" (p. 13). In brief, the process involved the following steps:

1. In depth identification and analysis of the factors influencing nebuliser adherence in PWCF through an examination of the existing literature, and a qualitative study in which participants viewed charts of their objective nebuliser adherence data within an interview about factors affecting their motivation, capability and opportunity to adhere to their nebuliser treatment (study 2.1). The Theoretical Domains Framework (TDF; [36]) which analyses Capability, Opportunity and Motivation in greater detail was used as a framework to guide the analysis.
2. Identification and evaluation of potential intervention functions (e.g. education, persuasion, enablement, environmental restructuring, modelling) to address the identified factors influencing nebuliser adherence in consultation with the research team, clinicians and PPI.
3. Development of intervention modules to include specific Behaviour Change Techniques to deliver intervention functions, selection of mode of delivery, and mechanism for tailoring of BCI delivery to meet individual needs with regard to Capability, Opportunity and Motivation. The module contents have been discussed and refined as a result of discussions with clinicians and PPI.
4. Identification of potential mediators of behaviour change, and identification of tools to measure each mediator.

The intervention arrived at through this process is described in Table 2.

Table 2: Intervention modules

Module	COM-B	Intervention functions	Behaviour Change Techniques	Mode of Delivery
Universal parts of the intervention				
**Self-monitoring**	Psychological capability<br>Reflective Motivation	Education<br>Environmental restructuring<br>Enablement	• Self-monitoring of behaviour<br>• Adding objects to the environment (CFHealthHub)	• Charts of objective adherence data presented within CFHealthHub
**Goal setting & review**	Psychological capability<br>Automatic motivation	Enablement<br>Incentivisation	• Goal setting (behaviour)<br>• Feedback on behaviour<br>• Discrepancy between current behaviour and goal<br>• Review behavioural goals<br>• Graded tasks<br>• Social reward	• Discussion and agreement of goal with interventionist<br>• Review of goal<br>• Feedback on progress (through CFHealthHub and interventionist)<br>• Visual reward if goal met on CFHealthHub
**Treatment plan**	Psychological capability<br>Physical Opportunity Social Opportunity<br>Automatic motivation	Training<br>Environmental restructuring<br>Enablement	• Action planning<br>• Habit formation<br>• Prompts/cues (tailored)	• Action planning tool within CFHealthHub<br>• Option to set reminders
**Confidence building**	Reflective Motivation	Persuasion	• Focus on past success	• Interventionist encouraging focus on periods of higher adherence on charts

Module	COM-B	Intervention functions	Behaviour Change Techniques	Mode of Delivery
<u>Tailored parts of the intervention (based on baseline COM beliefs and barriers questionnaire (COM-BMQ)[1] and consultation with interventionist)</u>				
**My treatment**	Reflective Motivation<br>Psychological capability	Education<br>Persuasion<br>Modelling	• Information about health consequences<br>• Credible source<br>• Salience of consequences<br>• Demonstration of the behaviour<br>• Vicarious consequences<br>• Self-talk	• Q&A linked to information within CFHealthHub (tailored by baseline beliefs and prescription data)<br>• Presentation though text, patient stories, 'talking heads' and animation<br>• Credible sources including clinicians, PWCF and interventionist<br>• Interventionist eliciting self-talk through focus on why motivation is not lower than rating given on pre-screening questionnaire
**Confidence building**	Reflective Motivation	Modelling<br>Persuasion	• Demonstration of behaviour	• 'Talking heads' videos of coping stories within CFHealthHub
**Problem-solving (including skills training)**	Physical capability<br>Psychological capability<br>Physical opportunity<br>Social opportunity	Training<br>Environmental restructuring<br>Enablement	• Instruction on how to perform the behaviour<br>• Demonstration of the behaviour<br>• Behavioural practice/rehearsal<br>• Problem solving<br>• Restructure the physical environment<br>• self-talk<br>• social support (practical)	• Tailored problem solving guided by interventionist<br>• Solution bank within CFHealthHub.<br>• Construction of if-then coping plans<br>• Videos demonstrating correct use of nebulisers within CFHealthHub

1 Incorporating the Beliefs about Medicines Questionnaire (BMQ-specific nebuliser treatment) Horne, 2010

6.4.2 Intervention providers

Interventionists may already be working at, or be new to participating organisations or be the ACtiF interventionist employed to deliver the trial locally at the site. Externally appointed staff will be recruited through a formal job interview. Suitable individuals will include registered nurses or other member of the multidisciplinary team or a ; graduate in a suitable subject such as psychology or, other relevant profession who holds relevant skills / experience. Candidates for the post will ideally have a minimum of two years postgraduate experience which might include delivering a research project to time and target. They will be employed on the Project to work to NHS Agenda for Change Band 4 or above. They must have access to a car for work purposes e.g. participant home visits.

Interventionists will be supported in the delivery of the intervention by members of the Multidisciplinary team (MDT) at the site in which they are based. MDTs will receive training about the approach of the intervention, and the way in which they can support its delivery (see page 28).

Training for interventionists in how to deliver the intervention according to the specifications of the behaviour change manual will be provided by Marlene Hutchings with oversight provided by Madelynne Arden and/or Judy Bradley. A comprehensive training manual and training programme will be developed to facilitate this. A certificate of competence will be provided prior to the interventionist being able to use CFHealthHub with participants.

An additional trained regional interventionist will offer support to trial sites. This on occasion will involve input to patients (face to face or telephone contact), and assisting with problem solving via liaising with the nebuliser company. They will be named on the local site delegation log.

6.4.3 Materials

The BCI contains two broad categories of components:

i. CFHealthHub behaviour change modules including adherence feedback used by PWCF and health professionals

ii. The behaviour change manual and toolkit used by the interventionist in interactions with PWCF in order to understand the specific barriers to adherence for that individual, and to tailor and personalise delivery of the behaviour change modules accordingly.

6.4.4 Procedures

The BCI will be delivered over a 4 to 6 month period through a combination of face-to-face sessions and contact via telephone with an interventionist, and through participant interaction with different modules of content available on CFHealthHub. The interventionist will discuss participant data with members of the MDT to ensure that care is informed by objective adherence data. If any concerns become apparent as the interventionists collect data and work with participants, these concerns will be passed onto the clinical team. The clinical team will

follow their standard procedures in relation to any concerns raised. The intervention content and delivery flow are outlined in Figure 5 and described below:

6.4.4.1 Consent Visit (all participants)

At the consent visit participants will be given a chipped nebuliser (eTrack) and Qualcom hub or the participants will receive a visit from a clinical trainer who will convert the participant's I-neb to a Bi-neb by adding a Bluetooth chip and providing a Smartphone hub. The clinical trainer may set up the Bi-neb in the patient's home or at hospital either during the main consent visit or at a separate visit after consent has been obtained. Both the eTrack and Bi-neb will connect to CFHealthHub which will enable adherence data be collected. The interventionist will input the participant's prescription details into CFHealthHub. Together these will allow the system to generate adherence charts for that participant. At this visit participants will complete a range of baseline measures (see Table 3) including the COM beliefs and barriers questionnaire (COM-BMQ) which will be entered into CFHealthHub. The responses to this questionnaire will be used to populate the 'My toolkit' section of CFHealthHub with specific tailored elements from the 'My treatment' modules prior to the Initial Intervention Visit. The participant's pseudomonas status will be clarified at baseline and confirmed by the PI with the opportunity to compare the participant's prescription with the pseudomonas status.

6.4.4.2 Initial Intervention Visit (intervention arm only)

Participants will be introduced to CFHealthHub. They will be asked to complete an online consent form on behalf of their NHS trust in which they will specify what additional data they would be willing for CFHealthHub to record and display (e.g. name, and uploaded photographs) and what functional options they would like access to (e.g. push notifications). Permissions may be changed at any time. The participant will have the option to upload their own "patient story" into CFHealthHub after completion of the online consent form.

The interventionist will discuss their motivation to adhere to their nebuliser treatment, will address beliefs associated with poor adherence and will refer back to answers on the COM-BMQ to elicit the participants beliefs associated with adherence. Participants will be shown 'My toolkit' which will have been prepopulated with tailored motivational content (see consent visit).

The interventionist and participant will look at and discuss the adherence charts on CFHealthHub with a focus on period of higher adherence. The interventionist will note any barriers raised by participants during this discussion.

The interventionist will support the participant to identify where and when additional nebuliser treatments could be fitted into their schedule and support them to make an action plan using the online tool available on CFHealthHub. This action plan will be saved to the 'My toolkit' zone. The interventionist will then agree a % adherence goal for the next four to six weeks based on the number of additional treatments that have been planned. This will be recorded on CFHealthHub and will be represented by a target line on the adherence charts.

If motivation is so low that participants are reluctant to set an action plan/goal then the interventionist will spend further time discussing motivation and will skip to confidence building (see below).

The interventionist will encourage participants to focus on likely problems or issues that might disrupt the achievement of the adherence goal and will use the Problem-solving module on CFHealthHub to address each of these anticipated problems. The Problem-solving module includes solutions based on educational content, practical support (e.g. model letters to

employers) and interactive tools. Relevant solutions will be saved to the 'My Toolkit' zone of CFHealthHub.
The interventionist will discuss the participant's confidence to meet their goal and will identify 2-3 'talking heads' videos showing other people with CF addressing and overcoming similar barriers to nebuliser adherence.
The visit will conclude with a review of the goal and the tailored and personalised contents saved to the 'My toolkit' zone of CFHealthHub. The interventionist will encourage a learning mindset, emphasising that even if adherence doesn’t increase starting to think about adherence will produce learning that will make subsequent attempts to change easier.

6.4.4.3 Participant Independent access to CFHealthHub (intervention arm)
Participants will have independent access to CFHealthHub at all times following the Baseline visit. They can, at any time, access their adherence charts, 'My toolkit' contents, and can browse the other areas of content as they wish. Frequency of access to each area of CFHealthHub will be monitored and recorded.
Adherence charts will provide colour -coded feedback about participant achievement towards their adherence goal so that they are provided with immediate, easy to recognise information about their achievements. Subject to consent, participants will be sent encouraging messages via push notifications, or alternatively when they access CFHealthHub, to match the progress made e.g. congratulations on achieving their goal, congratulations on having made progress towards their goal, encouragement to remember their action plan.

6.4.4.4 Review visit (Visit 3 - intervention arm)
At the review visit, the interventionist and participant will look at and discuss the adherence charts on CFHealthHub and goal achievement with a focus on progress made and periods of higher adherence.

If the adherence goal was met then the participant will be encouraged to set a new higher adherence goal or to a goal to maintain their current level of adherence which will be recorded on CFHealthHub. Following this the participant and interventionist will review the contents of 'My toolkit' and revise action plans, problems/solutions as required. If issues of motivation are still a concern the interventionist may recommend additional/alternate elements of content from 'My treatment' or 'Talking heads' to go into 'My toolkit'.
If the adherence goal was not met then the interventionist and participants will discuss the barriers to goal achievement (motivation, capability, opportunity). The interventionist will address beliefs associated with poor adherence and will add/revise the elements of content from 'My treatment' or 'Confidence building' to go into 'My toolkit'.
If no goal was previously set then the interventionist will review motivation and confidence and then will consider if the participant is ready to action plan and set a goal. If not they will spend more time reviewing motivation and confidence.
The participant will be encouraged to set a realistic % adherence goal for the next four to six weeks and this will be recorded on CFHealthHub. The interventionist will support the participant to revise their action plan as needed and save this to the 'My toolkit' zone. Based on the earlier discussion about the barriers that prevented goal achievement the Problem-solving module on CFHealthHub will be used to address each of the problems encountered, and any that are anticipated. Relevant solutions will be saved to the 'My Toolkit' zone of CFHealthHub.

The visit will conclude with a review of the goal and the tailored and personalised contents saved to the 'My toolkit' zone of CFHealthHub. The interventionist will re-emphasise a

learning mindset, emphasising that the participant cannot fail, but can learn from the process so that they can work together on the adherence challenge.

Participating centres will provide participants with contact details, typically telephone numbers, but other methods may be volunteered by centres. Contact details will be provided so that participants can contact the centre if they have queries or problems regarding CFHealthHub between visits. The interventionist will be able to feedback any information from the intervention delivery **after** the baseline intervention visit to members of the wider CF team. This may include adherence data from sessions with the participant's clinician and MDT particularly if the participant raises any concerns or issues e.g. side effects of a drug to allow their usual clinician to discuss this with them at their next clinic visit.

6.4.4.5 Subsequent Review (intervention arm)

Following these two sessions the amount of interaction which each PWCF has with the interventionist will be tailored to their needs and requirements although it is anticipated that these will normally marry with routine clinic visits: They may have additional face-to-face sessions or contact via telephone or e-mail. No more than one monthly face-to-face session will be conducted because of the research protocol; if the participant requests additional support, the centre may accommodate this at their discretion. Review meetings will take 30 minutes and be conducted over the 5month (+/- 1 month) of the follow-up period. The structure of review sessions will follow the same pattern as for 6.4.4.4.

6.4.4.6 Final research visit (5 months +/- 1 month from consent)

All participants will complete a final research visit 4-6 months from the date of consent. At this visit the interventionist will collect the primary and secondary outcome data (see table 3) including demography data, health care resource use and the participant completed questionnaires. At this final research visit the interventionist will re-check that all adherence data has been transferred to CFHealthHub. The eTrack can store approximately 6 months of treatment data, ensuring all the data is transferred at this visit should help to prevent missing data.

Following the final 4-6 month post-consent research visit, we will continue to collect: adherence data from CFHealthHub; exacerbations; FEV1 and ask participants the subjective adherence question until, 30th April 2017. At this point the study closes and the involvement of all participants ceases. After the trial ends (30/4/17), the aspiration is to allow participants in the control to have access to the intervention for which negotiations are ongoing. Currently funding is in place for the trial interventionists at study sites to deliver the intervention only over a 12-month period i.e. up to 30/4/17. It is anticipated that CFHealthHub used outside the trial would be delivered within the existing resources of the MDT so using CFHealthHub outside the trial should not need the trust to employ any additional staff members. As this is a pilot feasibility study where we are testing the intervention in participants, there is an expectation that further iteration of CFHealthHub may occur.

Figure 5. Behaviour change intervention flow chart

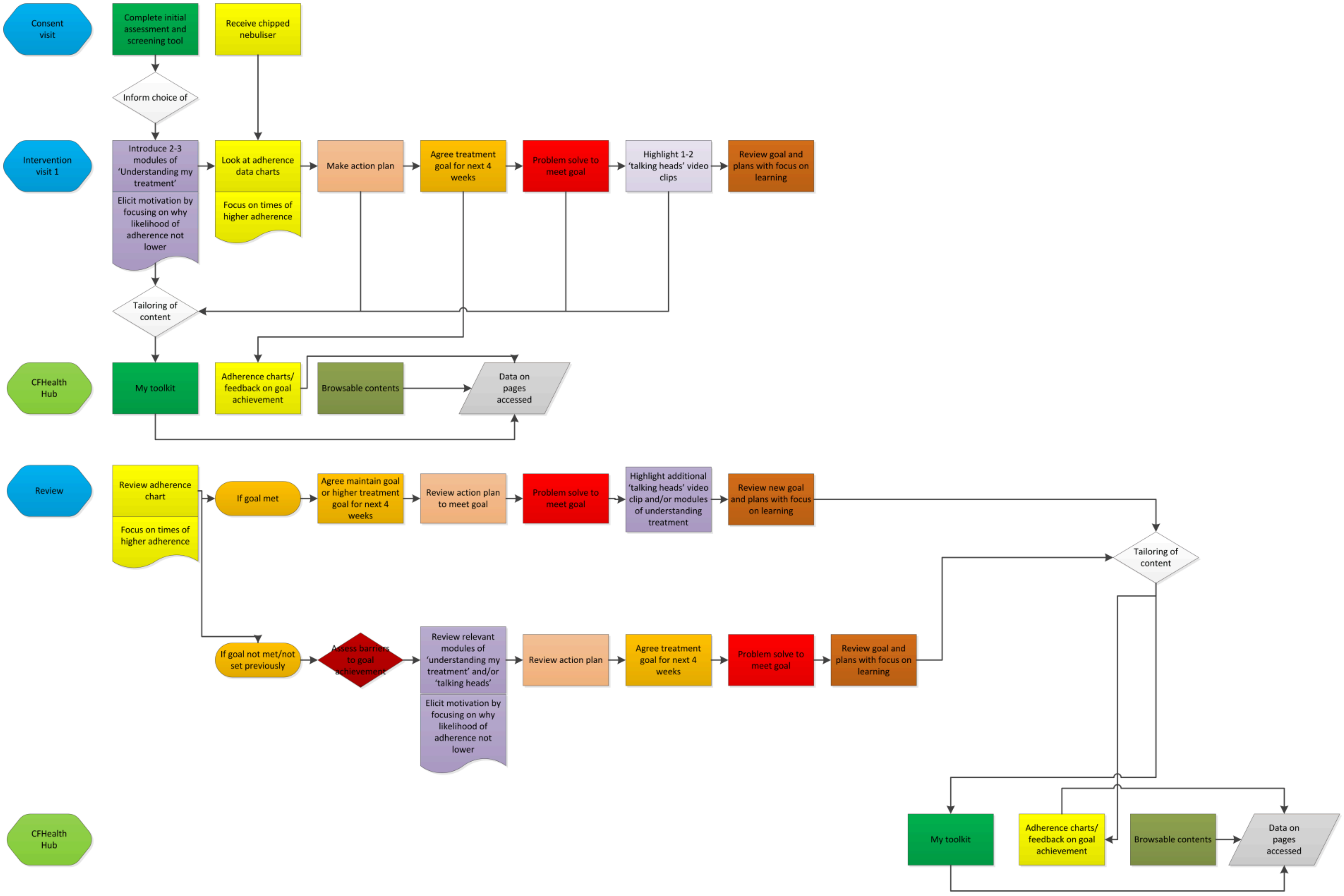

6.5 Usual care

Patients in both arms will receive usual care. Usual care is heterogeneous within and between centres, based on the needs of patients and the skills and interests of CF Unit staff. To better understand the configuration of usual care at participating centres a survey tool will be administered by the CTRU to the lead clinician at the centre. This will identify the spectrum of clinical and behaviour change interventions that are in use in the management and self-management of CF.

A minor component of the intervention is to train all members of the MDT in awareness of patient activation so that they are open to addressing issues raised for PWCF in the intervention arm. In addition, a staff member in the MDT will help to deliver the intervention. There is the possibility that the awareness of patient activation will have some effect on PWCF in both the intervention and control arms, and of leakage of the learning from the behaviour change component of the intervention to controls. We will investigate this possibility during the process evaluation.

Members of the MDT at each centre will receive one half-day, on-site, face-to-face training about the importance of objective nebuliser adherence data in the management of CF, and awareness of the importance of building patients' knowledge, skills and confidence to enable them to self-manage their treatment. This will include training in the interpretation of graphs and charts of objective adherence data produced by CFHealthHub, and the rationale for reducing target adherence in poor adherers in order to increase confidence. This will be delivered by designated members of the ACtiF research team.

Participants in the control arm will use a microchipped nebulizer but will not be able to access adherence data or other content and tools through CFHealthHub, neither will they receive the structured CFHealthHub intervention as described in the intervention manual. Control arm participants using Bi-neb nebulizers might have access to their data as part of routine care but this will not be in the user friendly format provided by the intervention.

One function of the qualitative research interviews with staff and control participants (see Section 8 below) is to understand the extent to which the patient activation awareness training has affected staff behaviour and whether control arm participants have received some aspects of the behaviour change intervention.

6.6 Criteria for discontinuing or modifying allocated interventions

There are no criteria for discontinuing treatment. Participants will be made aware that their participation is voluntary and they may discontinue study interventions, should they wish, at any time.

If a participant wishes to withdraw from treatment they will be able to speak to a member of the site study team i.e. ACtiF interventionist. This will be documented on a participant withdrawal form, within the Case Report Form. Any data already collected during the course of the trial up to the point of withdrawal will be used in the final analysis. We will ask the participants for their permission to continue to collect the primary outcome data i.e. CF exacerbations. The participant or clinician can make the decision to discontinue the allocated study intervention for any reason.

Participants will have the following options if they wish to withdraw:

1. Withdraw from the intervention i.e. intervention delivery visits only but will remain in the study. Patients can continue to use CFHealthHub. All study data would continue to be collected at subsequent follow up time points as per protocol.
2. Withdrawal from the study. Unless the patient objects, any data collected up to this point would be retained and used in the study analysis. The local interventionist would ask the participant if they agree to the collection of primary outcome data as defined in the protocol and or adherence data If

they agree to collection of adherence data, CTRU and or interventionist will continue to follow up participants for adherence data.

3. Withdrawal from the trial entirely. Unless the patient objects, any data collected up to this point would be retained and used in the study analysis. If the patient does not wish to be contacted with regard to primary outcome data or adherence data, no further contact with regard to the study will be made. If the participant does specifically request for all their data to be removed information regarding the participant will be retained at site, as part of the patient notes, along with their withdrawal form and request to delete the data.

A participant would be classed as complete if they have continued in the study until the last protocol defined visit, however there may be missing visits and / or data.

Loss to Follow-Up
A participant would be classed as lost to follow up if the participant has 1) not completed the study or 2) been withdrawn despite attempts for further contact, as per protocol, having been made. Unless the participant withdraws from the study entirely we will continue to collect the primary outcome data when possible (i.e. from medical notes).

This withdrawal section has been developed in accordance with the CTRU Participant Discontinuation and Withdrawal of Consent Standard Operating Procedure (SSU003).

6.7 Strategies to improve adherence to intervention protocols

6.7.1 For health professionals

The intervention protocols will be described in detail in an intervention manual. Interventionists will be trained to deliver the intervention according to the manual protocols. Interventionist training (as a form of behaviour change) will focus on Capability, Opportunity and Motivation. It will utilise evidence about the importance and likely effectiveness of the intervention and will challenge common misconceptions about adherence. Skills training and an introduction to the tools available on CFHealthHub will increase staff capability, and we will work with clinics and clinicians to ensure that the practical requirements for intervention delivery are in place: space, time etc (opportunity).
CFHealthHub will record interventionist access to the site. It will also automatize some of the tailoring of the intervention according to the COM-BMQ which will be completed online. The contents of 'My Toolkit' will be recorded for each participant so that we will have records of what content they have been recommended. Interventionists will also be required to complete session records each time that they deliver the intervention to record the decisions made and the reasons for these,

6.7.2 For patients

Where participants provide consent we will send optional push notifications to encourage engagement with CFHealthHub. For example, we will send congratulatory messages when adherence improves, encouraging messages to remind participants to engage with the content. Face-to-face visits will, where possible be arranged to coincide with clinic visits as per usual care, therefore minimising the additional burden on participants.

6.8 Relevant permitted / prohibited concomitant care

No concomitant care will be denied based on the research protocol.

7. Outcomes

7.1 Feasibility outcomes (‘stop-go’ or ‘success’ criteria for RCT)

In line with proposed CONSORT extension for pilot studies [37], in this section, we state the criteria for success of the external pilot trial. The criteria are based on the primary feasibility objectives, which provide the basis for interpreting the results of the external pilot and for determining the feasibility of proceeding to the full-scale study scheduled for months 31 to 60 of the project. Depending on the funder’s perspectives, the outcome of the external pilot might be:

(i) “Stop - main study not feasible”;
(ii) “Continue, but modify protocol - feasible with modifications”;
(iii) “Continue without modifications, but monitor closely - feasible with close monitoring”; or,
(iv) “Continue without modifications - feasible as is.”[37]

We anticipate that modifications to the research protocol will be necessary as the feasibility study progresses. Some of the qualitative research will be undertaken early in the pilot trial and lessons learned about the trial procedures will be identified and acted on during the pilot trial. There are three objective stop-go criteria:

1. Feasibility of recruitment to RCT
Defined as recruitment of no fewer than 48 participants randomised at two centres over four months, 75% of the rate required in the main trial;

2. Feasibility of retaining participants in the RCT
Defined as attrition from the research protocol of no more than 15% of randomised participants at 5 (+/-1) months.

If these are met the full trial will go ahead. If these are not met overall, but are met in the last half of the pilot trial after trial procedures have been improved based on lessons learned from the early stage of the pilot trial, then the full trial will go ahead.

7.2 Process data relating to the implementation of the trial

1. Number and characteristics of eligible patients approached for the study
Collected by centres in screening logs and transferred to Prospect database

2. Reasons for refused consent
Collected by centres in screening logs and transferred to Prospect database.

3. Reach
How many participants are consented into the study, sub-grouped by socio-economic status (from CF Registry), as a proportion of:

- Those approached, expressed quantitatively, based on ‘pre-screening’ logs completed by ACtiF interventionist;

- Those known to be eligible, expressed quantitatively based on CF Registry.

4. Participant attrition rate
Collected by centres in screening logs and transferred to Prospect database.

5. Reasons for attrition
Collected by centres in screening logs and transferred to Prospect database.

6. Maintenance:
The processes by which participants are kept involved in the collection of key secondary outcome data research data:

- The extent to which adherence data is successfully uploaded from the chipped nebulisers , described quantitatively using CFHealthHub (Intervention arm only).

7. Number of missing values/incomplete cases
Assessed by data management team, based on data in Prospect database.

8. Participant,/interventionist and members of MDT views on research protocols
Assessed through qualitative interviews and to include:

- Barriers to recruitment, problems encountered in reaching participants [38];
- Perceived problems with trial procedures such as recruitment, informed consent etc.
- Acceptability
- Perceived utility and burden of outcome assessments.

9. A survey on the content of usual care at participating centres
A CTRU staff member will complete this survey with the principal investigator, a senior medic or delegate working at the participating centre.

7.3 Process data relating to the implementation of the intervention

1. Context
Definitions of 'context' tend to cluster around setting, roles, interactions and relationships [39]. It is important that context is understood as diachronic and emergent rather than synchronic and static [40, 41]. Frameworks for process evaluation have defined 'context' as:

- "aspects of the larger social, political, and economic environment that may influence intervention implementation" [42];

- "factors external to the intervention which may influence its implementation, or whether its mechanisms of impact act as intended" [43].

The context, and its interaction with implementation, mechanisms of impact, outcomes, the description of the intervention and its causal assumptions [43] will be described using qualitative data from research interviews, field notes, study management logs, minutes and e-mails. The focus will be how the context of individual CF Units affects implementation of the intervention and its potential outcomes.

2. Implementation
Definitions of 'implementation' tend to cluster around the processes or stages of adoption, the methods, means or social organisation of bringing innovative practices into use [39]. One way of describing the process of getting research into practice is to use a process model [44]. To structure our narrative of how the complex intervention was implemented we will use a process model called the Quality of Implementation Framework [45].

3. Recruitment:
Based on e-mails and minutes we will describe in narrative terms, the procedures used to approach and attract to the project NHS Trusts and interventionists [42].

4. Training:
The comprehensiveness of the training component of the intervention for the health professionals delivering the intervention will be assessed by a combination of audio recordings of consultations and by interview.

5. Fidelity
"The extent to which the intervention was delivered as planned. It represents the quality and integrity of the intervention as conceived by the developers. Fidelity is a function of the intervention providers."[42]

- Interaction with participant along lines recommended by manual, determined by audio recordings of consultations between the interventionist and PwCF in the intervention arm.

- Recommendation of appropriate CFHealthHub tasks by interventionist, determined by audio recordings and by data from CFHealthHub;

The fidelity assessment will be developed and based on a tool used by Borelli et al [46].

6. Use [38] ***/ dose received*** [42] ***of intervention***
Use of CFHealthHub by participant, as proposed by interventionist, determined by data capture by CFHealthHub, including the online activities started and completed, minutes spent on recommended pages and which parts the participant has picked out and put in a "my favourites" page. The number of times, frequency over time and duration with which users log on to CFHealthHub, as well as the activities they perform while logged in, described quantitatively using data from CFHealthHub.
A record of the discussion between the interventionist and the MDT will be kept. This will include who was there, brief notes of what was discussed and any agreement of treatment goals made.

7. Acceptability
The acceptability of the intervention to hospital staff and PWCF assessed through semi-structured interviews.

8. Perceived benefits and harms
Assessed through semi-structured interviews with health professionals and PWCF.

9. Leakage of intervention to controls
Assessed through audio recordings of consultations between the MDT, interventionist, and PwCF in the control arm, and semi-structured interviews with PwCF in the control arm.

7.4 Clinical outcomes and covariates

The time schedule of enrolment, interventions (including any run-ins and washouts), assessments, and visits for participants can be found in Table 3 and Table 4 below.

7.4.1 Primary clinical outcome

The primary clinical outcome is the number of pulmonary exacerbations in 5 (+/-1) month post-baseline follow-up period, defined according to the Fuchs criteria [47]. An exacerbation of respiratory symptoms will be said to have occurred when a patient was treated with parenteral antibiotics for **any one of the following 12 signs or symptoms** [48]**:**

1. change in sputum;
2. new or increased hemoptysis;
3. increased cough;
4. increased dyspnea;
5. malaise, fatigue, or lethargy;

6. temperature above 38 °C;
7. anorexia or weight loss;
8. sinus pain or tenderness;
9. change in sinus discharge.
10. change in physical examination of the chest, derived from notes by site staff.
11. decrease in pulmonary function by 10 percent or more from a previously recorded value, derived from notes by site staff; or,
12. radiographic changes indicative of pulmonary infection, derived from notes by site staff.

The trial interventionist or prescribing clinician/nurse will collect data on the “exacerbations” form at the point of a participant starting a course of IV antibiotics.

7.4.2 Secondary clinical outcomes

1. **Body Mass Index (BMI).**

2. **Forced expiratory volume in 1 second (FEV_1):** standardised spirometry as a measure of condition severity [49].

3. **EuroQol EQ-5D-5L**: generic health status measure for health economic analysis [50].

4. **The Patient Activation Measure (PAM-13) (Health Style Assessment)**: assessment of patient knowledge, skill, and confidence for self-management [51]. *PAM-13 was labelled as “Health Style Assessment” following a request from the licence owners to ensure the purpose of the questionnaire is clear for participants.

5. **Assessment of routine :** measure of life chaos [52].

6. **Self-Report Behavioural Automaticity Index (SRBAI):** automaticity-specific subscale of the Self Report Habit index to capture habit-based behaviour patterns [53].

7. **Cystic Fibrosis Questionnaire-Revised (CFQ-R):** disease specific health-related quality of life instrument [54].

8. **The Patient Health Questionnaire depression scale (PHQ-8):** severity measure for depressive disorders [55].
9. **MAD (Medication Adherence Data-3 items) :** medication adherence measure

10. **The General Anxiety Disorder 7-item anxiety scale (GAD-7):** severity measure for anxiety [56].

11. **The Capability Opportunity Motivation Behaviour Beliefs Questionnaire (COM-BMQ):** This questionnaire incorporates:

    a. **The Beliefs about Medicines Questionnaire - specific (Nebuliser adherence) (BMQ 21-item):** a validated self-report tool[57], customised by the author to identify perceived necessities and concerns for nebuliser treatment.
    b. The following project-specific items: one additional belief item, one intention item, one confidence item, and a list of barriers. These will serve as a tailoring tool for the intervention and also as a secondary outcome measure.

12. **Subjective adherence single question:** self-report estimate of adherence as a percentage. Self-reported problems: identification of capability and opportunity barriers to nebuliser adherence

13. **Concomitant medications:** bespoke instrument, designed for this research project.

14. **Resource use form:** interventionist collects data from a combination of hospital notes and the NHS patient electronic system to determine 1) inpatient IV days 2) Routine clinic visits 3) Unscheduled outpatient contacts 3) unscheduled inpatient stays.

15. **Exploratory analysis of habit formation**: analyses with the objective nebuliser data will be performed to explore the process of habit formation with the delivery of the adherence intervention

16. **Prescription**: a monthly prescription check to both check for data transfer to CFHealthHub and review for an indication that the prescription has changed or indication of microorganism e.g. pseudomonas (please see table 2 and 3 and refer to section 10.1.1).

17. **Adherence to prescribed medication (see 7.4.3)**

18. **Any treatment with IV antibiotics**

7.4.3 Adherence to prescribed medication

Adherence to prescribed medication will be defined in several ways including:

1. Unadjusted adherence
2. Simple normative adherence (without numerator adjustment)
3. Sophisticated normative adherence (without numerator adjustment)
4. Simple normative adherence (with numerator adjustment)
5. Sophisticated normative adherence (with numerator adjustment)

Further detail about the outcomes will be reported in the trial statistical analysis plan.

Table 3. Individual-level data derived from PWCF and sites

	Where?	Completed by?	Consent visit	Baseline (intervention) visit	At clinic visits	Exacerbations episode	5 months (+/- 1 month) from consent visit	Up to 30th April 2017
**Enrolment**								
Pre-screening form (before 1st visit)	Prospct	Site	-	-	-	-	-	-
Confirmation of eligibility form	Prospct	Site	●	-	-	-	-	-
Informed consent	Prospct	Site	●	-	-	-	-	-
Intravenous days in last registry year	Prospct	Site	●	-	-	-	-	-
Pseudomonas status +	Prospct	Site	●	-	-	-	-	-
**Primary outcome**								
Exacerbations form including:<br>Parenteral antibiotics<br>Change in sputum*<br>New or increased hemoptysis*<br>Increased cough*<br>Increased dyspnea*<br>Malaise, fatigue, or lethargy*<br>Temperature above 38 °C*<br>Anorexia or weight loss*<br>Sinus pain or tenderness*<br>Change: sinus discharge*<br>Change: phys. exam. chest*<br>Decrease: pulmonary function *<br>Indicative radiographic changes*	Prospct	Site	●	-	-	●	●	●
**Secondary outcomes**								
BMI (height and weight)	Prospct	Site	●	-	-	-	●	-
FEV_1	Prospct	Site	●	-	●	-	●	●
EQ-5D-5L**	Prospct	PWCF	●	-	-	●	●	-
PAM-13(Health Style Assessment)	Prospct	PWCF	●	-	-	-	●	-
Assessment of Routine	Prospct	PWCF	●	-	-	-	●	-
SRBAI	Prospct	PWCF	●	-	-	-	●	-
CFQ-R	Prospct	PWCF	●	-	-	-	●	-
PHQ-8	Prospct	PWCF	●	-	-	-	●	-
GAD-7	Prospct	PWCF	●	-	-	-	●	-
MAD-3 (Medication Adherence Data-3 items)	Prospct	PWCF	●	-	-	-	●	-
COM-BMQ	Prospct	PWCF	●	-	-	-	●	-
Objective adherence	CFHH	CFHH	●	-	●	-	●	-
Subjective adherence single question	Prospct	PWCF	●	-	●	-	●	●
Concomitant medications	Prospct	Site	●	-	-	-	●	-
Other SAEs	Prospct	Site	-	-	●	-	●	-
Resource use	Prospct	Site	-	-	-	-	●	-

+ Pseudomonas (or other microorganism) status will be checked together with the monthly prescription
* Only required where PWCF indicates they have received parenteral antibiotics

** EQ5D-5L collected at the start and end of every exacerbation episode

Table 4. CFHealthHub data (research arm only)

	Completed by?	Baseline (intervention) visit	At intervention visit s with interventionist	Between sessions	At clinic visits	5 months (+/- 1 month) from consent visit	Up till 30th April 2017
**Clinician metrics**							
Adherence data*	PWCF	●	●	●	●	●	●
Recommendation of modules by interventionist	Interventionist	●	●	-	●	-	x
Feed back to participant their adherence data screens (data click)	Interventionist	●	●	-	●	-	x
Check prescription with participant	Interventionist	●	●	-	●	-	x
Order of clicks	CFHH	●	●	-	●	-	x
Interventionist responds to patient changing prescription	Interventionist	-	●	●	●	●	x
Monthly check on prescription +	Interventionist / CTRU	●	●	●	●	●	x
Time in and out preparation	Interventionist /CFHH	●	●	-	-	●	x
Time in and out with patient	Interventionist /CFHH	●	●	-	-	●	x
Time in and out review	Interventionist /CFHH	●	●	-	-	●	x
**Patient metrics**							
Adherence (number of nebulized doses taken per day.) [1]	PWCF	●	●	●	●	●	x
Duration of inhalation	Nebuliser	●	●	-	-	-	x
Accessing CFHealthHub – look at adherence data	PWCF	●	●	-	-	-	x
Accessing CFHealthHub – look at 'My Toolkit'	PWCF	●	●	-	-	-	x
Accessing CFHealthHub problem solving / education / talking heads pages outside of 'My Toolkit'	PWCF	●	●	-	-	-	x
Accessing CF HealthHub – first to last click in a session	PWCF	●	●	-	-	-	x

*Adherence data collected for both research and control arms
+ Monthly prescription checked by CTRU centrally to alert local interventionists to any potential changes in control arm and potentially also intervention arm
X data continued to be collected in CFHealthHub and interventionist responds for those participants who have "opted in" to receive intervention till 30/4/17
[1]To be broken down in statistical analysis plan.

Figure 6. Participant timeline for the external pilot RCT

Enrollment

Identification and approach, by letter, following up with phone call (pre-Consent form) :

1) PWCF identified through the CF registry;

2) Screened for eligibility (pre-screening tool):

Inclusion: PWCF who have data within the CF registry aged > 16 years & taking inhaled mucolytics or antibiotics via a chipped nebuliser (e.g eTrack or I-Neb) or able and willing to take via eTrack or I-Neb.

Exclusion: PWCF who are post-lung transplant or on the active lung transplant list, patients receiving palliative care, with palliative intent, for whom trial participation could be a burden, patients lacking in capacity to give informed consent or using dry powder devices to take antibiotics or mucolytics.

3) Direct care team 1) Sends potential participant PIS and introductory letter by post and Interventionist follow's up by phone within 1 week to ensure they have received the information and allow an opportunity for questions AND/OR **2)** Direct care team gives patients written information during routine clinic visit and asks permission to follow up the patient with a phone call to answer further questions and discuss involvement.

↓

CONSENT VISIT, at home or in clinic:

1) **Screening form completed** (confirmation of eligibility)
2) **Informed consent.**
3) **Data collection:** Intravenous days in last registry year; BMI; FEV1; EQ-5D-5L; PAM-13; CHAOS; MAD-3; SRBAI; CFQ-R; PHQ-8; GAD-7; COM-BBQ;Exacerbations form; Concomitant medications; subjective adherence question
4) **Participant given data logging nebuliser and Qualcomm hub.***

↓

Allocation

Randomisation (n~64):
ACtiF interventionists randomises using web-based system between consent and baseline visits and informs participant ideally within 1 week of consent visit.

Intervention arm
Research: Monitoring and feedback (n=32):
Initial intervention visit: at home or in clinic (2-6 weeks from consent visit) (1 hour)

1) Adherence data uploaded, fed back and reasons for suboptimal adherence discussed in order to identify problems to solve using the Adherence Support Process;
2) Intervention chosen based on barrier from "library" in the Adherence Support Process;
3) Supportive contact offered via phone or email; and,
4) Participants given personal access to cfhealthhub data.

Manualised intervention delivered by trained researcher in initial intervention visit and subsequent visits normally every 4-6 weeks from this initial visit till six month visit (see below)

Control arm
Usual care n=32:
Adherence data uploaded. Care team and PWCF remain blind to adherence data.

Follow-up

ACtiF Researcher collects exacerbation data
Data collected: Exacerbations form triggered by commencement of IV antibiotics; EQ5D-L at both start and end of exacerbation episode

Routine Clinic Visits:
Data collected from all routine clinic visits after the date of consent.
Data collected by ACtiF researcher except where specified: (i) FEV1; (ii) subjective adherence question (iii) exacerbations form.
Adherence data continued to be uploaded to CFHH.

Monthly follow up by CTRU researcher and/or ACtiF interventionist
Monthly check of adherence data and contact with participant where missing adherence data or requirement to follow up on changes to prescription

↓

5 month (+/- 1 month) visit
Primary clinical outcome: Number of exacerbations (Fuchs criteria)
Secondary outcomes: BMI; FEV1; EQ-5D-5L; PAM-13; CHAOS; MAD-3; SRBAI; CFQ-R; PHQ-8; GAD-7; COM-BBQ;Exacerbations form; Concomitant medications; Other SAEs, resource use questionnaire

*When I-nebs are converted to Bi-nebs a representative from the company (Philips) will do this between the consent visit and first intervention visit.

The study recognises that flexibility in accommodating participant schedules may cause time windows to change but this will allow us to adapt the intervention for the main RCT.

8. Sampling

8.1 Quantitative components

8.1.1 Sites

Two large specialist CF centres have been screened for their ability to recruit participants based on the number of participants they have on their CF registry and their motivation to participate in the pilot trial.

8.1.2 Sample size

The sample size for a feasibility study should be adequate to estimate the uncertain critical parameters (standard deviations for continuous outcomes; consent rates, event rates, attrition rates for binary outcomes) needed to inform the design of the full RCT with sufficient precision [37, 58–60]. For the main RCT, the target sample size is 688 participants (344 per arm). We are proposing that 15 CF units recruit on average 46 patients in six months, a recruitment rate of approximately eight patients per centre per month.

To assess whether this recruitment rate is feasible the external pilot RCT will open in two CF units for 12 months, with four months recruitment, one months 'run-in' period (the period between the consent and baseline visit), and 5 (+/-1) months follow up. To match the proposed recruitment rate of the main RCT, the target sample over the four months for which the pilot RCT is open, will be 32 per centre (64 in total from the two pilot centres). We propose to recruit to time, that is for a fixed period of four months rather than to a fixed sample size. We would want to see a minimum of 75% of the recruitment target to be confident of the trial viability i.e. at least 48 patients in total consented and randomized in four months' of recruitment from two centres.

8.1.3 Approach, non-participation and recruitment

Approach: Health professionals involved in approaching and screening PWCF and collecting data will be trained in the study protocol and procedures. Additionally those taking consent will have up-to-date training in Good Clinical Practice (GCP). All study personnel will be named on the study delegation log. Health professionals working with the CF team will identify a sample of PWCF registered at the centre via the CF registry database locally. All inclusion and exclusion criteria will be assessable via patient records and they will exclude any patients who do not fit the eligibility criteria.

A member of the participant's direct clinical team will send the potential participant a PIS and introductory letter by post or give the written information during a routine clinic visit. A sticker with a website address and Quick Response code will be placed in the envelope both of which will link to a video of the researcher explaining the study. If information is provided in a routine clinic visit, the clinical care team will seek permission for the ACtiF Interventionist to follow up with a phone call in order to answer any further questions and discuss involvement. Written informed consent may be conducted at this visit where the participant is happy to take part as this is a low risk trial.

Telephone call: Up to a week after posting out the information, the ACtiF Interventionist will telephone the PWCF to discuss the study over the phone and answer any questions. If the potential participant is happy to take part, the ACtiF Interventionist will arrange an appointment to gather written informed consent.

Non-participation: Spontaneously offered reasons for non-participation in the trial will be recorded.

8.2 Qualitative components

<u>*At each of the two pilot sites*</u> we will undertake:

- Audio-recordings of all 16 initial assessments for PWCF in the intervention arm and 10-12 consultations between the senior interventionist from the MDT (or other MDT member) and PWCF in the control arm. Numbers will depend on numbers of PWCF giving written consent for this.

- 10-12 semi-structured face-to-face (or telephone or skype) interviews with PWCF receiving the intervention and 10-12 semi-structured face-to-face interviews with PWCF in the control arm (total n~40-48 PWCF; n~40-48 interviews);

- two semi-structured face-to-face (or telephone or skype) interviews with each of the two interventionists in each centre (total n=4 interventionists; n=8 interviews); and,

- two semi-structured (face to face, telephone or skype) interviews with two members of the MDT (total n=4 staff; n=8 interviews).

Written informed consent will be obtained from both the interventionist and the PWCF participating in the audio recording when they consent to be in the study. Separate consent will be sought from PWCF and interventionists or members of the wider CF team for semi-structured interviews.

9. Assignment of interventions

9.1 Sequence generation

Participants will be allocated in equal proportions to one of the two groups using a computer generated pseudo-random list, stratified by centre and the number of days participants have been on IV antibiotics in the previous 12 month period as collected at consent visit, with random permuted blocks of varying sizes. The two categories for stratification within the number of IV days will be (i) less than or equal to 14 days and (ii) greater than 14 days.

9.2 Allocation concealment

The allocation sequence will be hosted by the Sheffield CTRU in accordance with their standard operating procedures and will be held on a secure server. Access to the allocation sequence will be restricted to those with authorisation. The sequence will be concealed until recruitment, data collection, and analyses are complete.

9.3 Implementation

The allocation sequence will be created by a Sheffield CTRU statistician who is not otherwise associated with the trial. At the consent visit, a health professional who is named on the delegation log, will go over the patient information sheet again with the study candidate and answer any questions. If the PWCF is still willing to enter the trial, they obtain full written consent and complete the eligibility form. If the participant is eligible, then baseline assessments will be taken. The recruiting health professional will log into the remote, secure Internet-based randomisation system and enter basic demographic information, after which the allocation will be revealed.

9.4 Blinding

After revelation of the allocation, only the statisticians will be blinded to allocation as per CTRU SOPs (ST001 and ST005)

10. Data collection, management and analysis

10.1 Quantitative data

10.1.1 Data collection methods

Data handling and record keeping. The Sheffield CTRU will oversee data collection, management and analysis and ensure the trial is undertaken according to Good Clinical Practice Guidelines and CTRU standard operating procedures. Data will be collected and retained in accordance with the Data Protection Act 1998. Patients will be reassured that all data which are collected during the course of the research will be kept strictly confidential.

The study team will train those collecting data in the study procedures before the trial begins. Data will either be collected directly from the participants, carers, interventionist, CFHealthHub or from source documents (e.g. patient notes) and input onto the CRF or Sheffield CTRU's electronic web-based data capture system (Prospect). The Data Monitoring and Management Plan for the study will provide further guidance on the types and levels of data and how these will be monitored and verified. Some essential documents may be posted to the central team to facilitate this e.g. participant consent forms in which case this will be detailed in the appropriate participant PIS and consent forms.

The CTRU will perform checks with the participant via monthly phone calls to ensure data is being captured and alert the local interventionist if there is an indication of a prescription change and a need to check pseudomonas (or other microorganism) status. This is required for the correct denominator to assess "normative adherence". Data will be extracted from the CF registry to understand exacerbations in the preceding 12 months since prior exacerbations can have a bearing on the optimum target regimen.

Plans to promote participant retention and complete follow-up.

Participant retention will be ensured by the following procedure:

1. At each point of contact, the interventionist will check with the participant that the Qualcomm hub or Smartphone hub is plugged in and turned on. A member of CTRU who is performing data and prescription checks may alert the interventionist. They will remind the participant of the proximity required for data transfer (10 metres)
2. In the event of no data being displayed in CFHealthHub for a period of at least a week (and the participant is not known to be on holiday) the interventionist will make contact with the participant (Email/Text/Telephone call) to check that the following
   - That the Qualcomm or Smartphone hub is plugged in
   - That the Qualcomm hub is working (showing solid green and yellow lights on the display)
   - That they have been within range of the Qualcomm hub sufficient to facilitate data transfer (10 metres)
   - That the Smartphone hub is switched on (showing the locked 'password' screen when any button is pressed)
   - That the Bi-neb and Smartphone hub have been kept in the same room, or at least have been in close proximity at some point during the day.

Any participants using the Bi-neb who are still experiencing issues after following the steps above, may receive a face to face or telephone support (at home or hospital) from the clinical trainer to resolve any outstanding issues.

Troubleshooting:

Data capture will be monitored both by interventionist at the site and centrally by the CTRU. In the event of data not being uploaded patients will be contacted to trouble shoot problems. Patients will be offered support to suit their circumstances including home visits (conducted by the members of the site research team) where necessary.

10.1.2 Data Management

Anonymised trial data will be entered onto a validated database system designed to an agreed specification between the Chief Investigator and Sheffield CTRU. The research staff at sites (mainly the ACtiF interventionist) will be responsible for data entry locally. The Sheffield CTRU Trial Manager, research assistant and the Data Management Team will work with sites to ensure the quality of data provided. The study manager, research assistant, data manager, PI's, any research nurses and site interventionist will have access to the anonymised data on the database through the use of usernames and encrypted passwords. The system has a full electronic audit trail and will be regularly backed up. The secure data management system will incorporate quality control procedures to validate the study data. Error reports will be generated where data clarification is needed. Output for analysis will be generated in a format and at intervals to be agreed between Sheffield CTRU and the Chief Investigator.

Trial documents will be retained in a secure location during and after the trial has finished. The study will use the CTRU's in-house data management system (Prospect) for the capture and storage of participant data. Prospect stores all data in a PostgreSQL database on virtual servers hosted by Corporate Information and Computing Services (CiCS) at the University of Sheffield. Prospect uses industry standard techniques to provide security, including password authentication and encryption using SSL/TLS. Access to Prospect is controlled by usernames and encrypted passwords, and a comprehensive privilege management feature can be used to ensure that users have access to only the minimum amount of data required to complete their tasks. This can be used to restrict access to personal identifiable data.

Participants who give consent to the qualitative part of this study will also give consent to their name and address to be given to the University of Sheffield qualitative research staff in order to be contactable.

10.1.3 Data quality assurance

Prospect provides a full electronic audit trail, as well as validation and verification features which will be used to monitor study data quality, in line with CTRU SOPs and the Data Management Plan (DMP). Error reports will be generated where data clarification is required. Rates of missing data and data points which are out of the expected or allowed range will be presented to the team at monthly management group meetings.

10.2 Qualitative data

10.2.1 Audio recordings of consultations

All initial assessments will be audio recorded with permission (n=16 in each site). Findings from early assessments will be fed back to the interventionist so that changes can be made to the intervention delivery before subsequent assessments. Consultations between the senior interventionist and PWCF in the control arm will be audio recorded with permission (n=10-12 in each site). Encrypted digital recorders will be used and recordings sent securely to the research team for analysis.

10.2.2 Semi-structured interviews: participants

In each site we will interview 3-4 PWCF receiving the intervention who are recruited at the beginning of the pilot. We will interview them around one month into the intervention to seek views of the most intensive part of the intervention. This will identify any problems early and be fed back to the intervention development team, staff delivering the intervention, and trial staff. We will interview 5-6 PWCF around four to six months into the intervention. These PWCF will have experienced more independent use of the CFHealthHub and we can explore how to keep PWCF engaged with the intervention in the longer term. We will interview 2-3 PWCF who drop out of the intervention to explore why this occurred. We will interview 10-12 PWCF in the control arm around four to six months into the trial to explore whether they have experienced aspects of patient activation and leakage of the intervention.

10.2.3 Semi-structured interviews: professionals

The first interviews with the interventionist and senior interventionist in each site will take place after they have undertaken assessments with the first few PWCF to identify teething problems with the intervention or the trial and the comprehensiveness of the training sessions they received. The findings will be fed back to the team to consider whether changes are needed to the intervention or trial protocol. The second interviews will take place when the first few PWCF have completed the intervention to allow the interventionist to reflect back over the whole process. The interventionists may have different lengths of experience of working with CF, nebulisers or behaviour change and we will consider the influence of differences in backgrounds on their ability to implement the intervention.

We will also undertake interviews with two members of the MDT at each centre when the first few PWCF have received 2-3 months of the intervention and then again towards the end of the feasibility study when all PWCF have been recruited and received around 3 months of the intervention.

10.2.4 Undertaking the interviews

For the interviews we have developed topic guides based on our research questions and these are attached to the application. Topic guides develop throughout any qualitative interview study and our topic guides may change as the study progresses. We will audio record all interviews after receiving written permission to do so. We will use an encrypted digital recorder. Reflexive notes will be made during and after the interviews. We expect

interviews to last around one hour. We do not expect data saturation in pilot studies; the aim is to identify any learning that can be addressed in preparation for the full trial.

11. Data analysis

11.1 Quantitative analysis

The analysis will be performed after data lock by a CTRU statistician under the supervision of the senior study statistician. As the trial is a pragmatic parallel group RCT data will be reported and presented according to the CONSORT 2010 statement [61] with reference to proposed extension for pilot / feasibility studies [37]. As a pilot/feasibility study the main analysis will be mainly descriptive and focus on confidence interval estimation and not formal hypothesis testing [58]. We will report rates of consent, recruitment and follow-up by centre and by randomized group.

Clinical outcome measures will be summarised overall and by randomized group. Baseline demographic (age, gender), physical measurements (e.g. weight, height, BMI), and patient reported outcome measures (EQ-5D, PAM-13, Assessment of Routine, MAD-3, SRBAI, CFQ-R, GAD-7, COM-BMQ, PHQ-8), and clinical measurements (e.g. FEV1, IV days in last registry year) will be described and summarised overall and for both treatment groups.

The primary outcome is the number of pulmonary exacerbations treated with IV antibiotics over the 6 month post-randomisation follow-up period. We will also include, as part of the feasibility analysis, estimation of the effect size for the 6-month pulmonary exacerbations outcome with 95% confidence interval estimates to check that the likely effect is within a clinically relevant range (as confirmation that it is worth progressing with the full trial). For this we will use a Poisson generalised linear model (GLM). Secondary continuous outcomes such as six-month post randomisation FEV1, BMI EQ-5D, PAM-13, Assessment of Routine, MAD-3, SRBAI, CFQ-R, GAD-7, COM-BMQ, PHQ-8) will be analysed with a multiple linear regression model with the baseline value of the outcome and randomised group as covariates. The treatment group coefficient and its associated 95% confidence interval will be reported from the various multiple linear regression models. The mean level of adherence (to prescribed medication) between the intervention and control groups over the 6 month post-randomisation follow-up period will also be reported and compared between the groups and a 95% confidence interval (CI) for the mean difference in this parameter between the randomised groups will also be calculated.

Further analyses with the objective nebuliser data will be performed to explore the process of habit formation with the delivery of the adherence intervention. The analyses will include:

(a) generating objective habit scores by taking into account time of nebuliser use

(b) using statistical process control to identify when periods of stability is achieved

(c) other time-series methods, including cross-correlation between habit scores and adherence.

Adverse events will be based on serious adverse events (SAE) case report forms. A serious adverse event is defined as any adverse event or adverse reaction that results in death, is life-threatening, requires hospitalisation or prolongation of existing hospitalisation, results in persistent or significant disability or incapacity, or is a congenital anomaly or birth defect.

The following summaries will be presented as overall rates and stratified by AE classification:

- the number and percentages of patients reported as having Serious Adverse Events (SAE) in each treatment arm; and,

- the number and percentages recorded as having all forms of Adverse Events (AE) in each arm.

This information along with the acceptability of the study design and protocol to patients/GPs; the safety of the intervention; patient recruitment and attrition/retention rates will enable us to determine whether or not the definitive RCT is feasible within a satisfactory timescale and cost envelope using UK centres alone.

11.2 Qualitative analysis

Transcripts will be coded using the latest version of NVivo (QSR International). The analysis will use the National Centre for Social Research 'Framework' approach [62]. AO'C and SD will undertake the following stages of the analysis of patient transcripts: familiarisation; identifying a thematic framework; indexing; charting; and, mapping and interpretation. The theoretical framework for understanding intervention adherence is the Necessities-Concerns framework [63] within the COM-B system [18]. This will be used within the thematic framework. We will use the process evaluation functions of context, mechanisms and implementation to frame the analysis [43]. Within mechanisms we will use the COM-B system as stated above and consider the use of the Theoretical Domains Framework [36]. We will compare and contrast findings from each site because the different backgrounds of the interventionists, and the different contexts in which care is provided in each CF unit, may affect implementation and acceptability of the intervention.

Figure 7. Assumptions of the MRC Guidance on Process Evaluation

[39, 64]

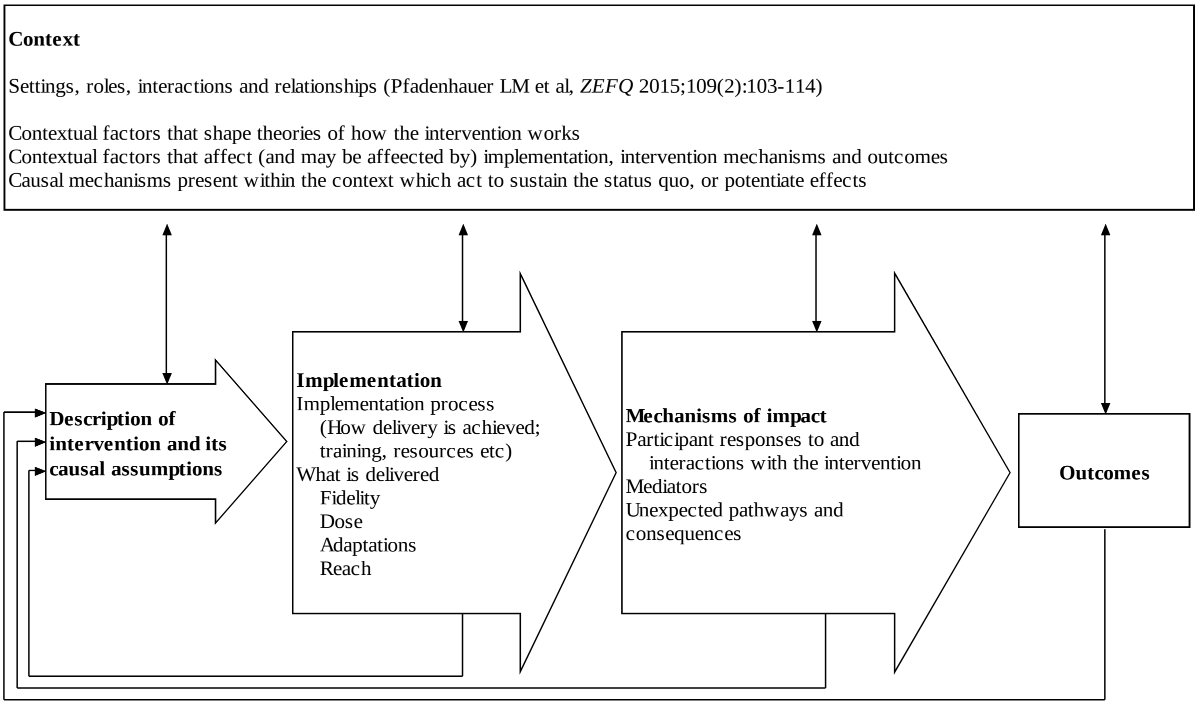

This qualitative research will:

- Inform the refinement of the intervention (e.g. CFHealthHub, training of interventionists, initial assessments, manualised instructions) and its implementation (e.g. introduction within a CF Unit) for use in the full trial.
- Inform refinement to trial procedures for the full trial.
- Inform the selection of the final secondary measures used in the full trial to ensure they address the perceived benefits of the intervention.
- Help to understand the extent of any leakage of the intervention to controls.

11.3 Combining data and findings from the different components

We will use Farmer's triangulation protocol to display the findings from each component of the study together and discuss as a team the extent to which findings converge, complement each other or contradict each other [65, 66]. For example, we will display all findings about recruitment together to consider the feasibility of recruitment for the full trial and the actions required to ensure feasibility. We will also display in a matrix the qualitative and quantitative data for individual PWCF who have received the intervention and been interviewed [66]. We will use this to consider the extent to which our secondary outcome measures identify issues raised by PWCF in the interviews.

12. Monitoring

12.1 Oversight

The CTRU SOP GOV003 Data Monitoring and Ethics Committee states "A DMEC does not need convening in studies that carry low risk to patients". This project involves

delivering a behaviour change intervention through the website CFHealthHub and would therefore be classified as low risk.

The overall responsibility for the study will be with Sheffield Teaching Hospitals NHS Trust who will act as sponsors for the study. The local Principal Investigator (PI) will be responsible for the study at each participating site and it will be registered and approved with each local R&D department. The study will be conducted in accordance with the protocol, GCP and Sheffield CTRU Standard Operating Procedures. The two committees which will govern the conduct of the study are:

1. Programme Steering Committee (PSC)
2. Project Management Group (PMG)

The PSC will be responsible for the overall conduct of the trial and consists of an independent chair and four other independent members including a statistician and PPI representative. The committee will meet every 6 months to monitor the study.

The PMG will comprise of the trial manager and the core research team . The PMG will meet on a monthly basis to monitor the day-to-day running of the trial. The Trial Manager will be jointly supervised by the CI and the Assistant Director of CTRU via the form of regular meetings (face to face and telephone calls). The Trial Manager will be responsible for liaising with the whole project team. Trial monitoring procedures will be assessed based on the level of risk of the study. The Site Monitoring Plan will outline the types and frequency of site monitoring activities for the study and this will be agreed with the Sponsor prior to the start of the study.

12.2 Description of any interim analyses and stopping guidelines

There are no planned interim analyses or stopping guidelines for this study.

12.3 Harms (safety assessments)

12.3.1 Serious Adverse Events

Trial sites are to report Serious Adverse Events (SAEs) in conjunction with the CTRU standard operating procedure PM004 (Adverse events and serious adverse events). The definition of an SAE is as follows:

- results in death;
- is life-threatening* (subject at immediate risk of death);
- requires in-patient hospitalisation or prolongation of existing hospitalisation;**
- results in persistent or significant disability or incapacity;
- consists of a congenital anomaly or birth defect; or,
- is another important medical event that may jeopardise the subject.***

* ‘life-threatening’ refers to an event in which the patient was at risk of death at the time of the event; it does not refer to an event which hypothetically might have caused death if it were more severe.
**Hospitalisation is defined as an inpatient admission, regardless of length of stay, even if the hospitalisation is a precautionary measure for continued observation. Hospitalisations

for a pre-existing condition, including elective procedures that have not worsened, do not constitute an SAE.
***Other important medical events that may not result in death, be life-threatening, or require hospitalisation may be considered a serious adverse event/experience when, based upon appropriate medical judgment, they may jeopardise the subject and may require medical or surgical intervention to prevent one of the outcomes listed in this definition.

It is not anticipated that there will be many SAEs related to the behaviour change intervention. We will report any SAEs which are deemed related to the trial intervention and unexpected to the Sponsor within the specified timeframes below (12.3.4).

12.3.2 Adverse events we require reporting:

We do require that sites report any new diagnosis of depression which requires treatment with medication or psychological therapy e.g. Cognitive Behavioural Therapy (CBT).

12.3.3 Expected SAEs and adverse events

Certain adverse events are common to CF and associated medications. Expected SAEs must be reported in the annual safety report. Hospitalisation as a result of an exacerbation will be recorded in the study database and not be reported as an SAE.

Expected AEs in relation to medications or common in patients with CF

1. Acute FEV1 drop >15% after 1st dose of medication
2. Increased productive cough
3. Nasal congestion or stuffy nose
4. Chest congestion
5. Wheezing
6. Chest pain or chest discomfort
7. Voice alteration/change
8. Dysponea (breathlessness)
9. Haemoptysis (coughing blood)
10. Rhinitis
11.
12. Headache
13. Crackles in lung
14. Throat irritation/ sore throat
15. URTI
16. Sinusitis
17. Deafness
18. Indigestion / reflux
19. Tonsillitis
20. Joint pain
21. Decreased appetite
22. Fatigue
23. Headache

24. Distal intestinal obstructive syndrome
25. Fever
26. Otitis media or ear infection
27. Conjunctivitis
28. Pneumothorax
29. Decreased exercise tolerance
30. Pyrexia
31. Abdominal pain
32. Influenza
33. Pseudomonas infection
34. Vomiting
35. Diabetes
36. Pneumonia

12.3.4 Reporting

Adverse events and SAEs can be reported for participants at any stage of their trial participation. A member of the site study team (interventionist, clinician or other) will enquire about any adverse events at routine clinic appointments. These will be record on the adverse event section of the paper CRF and database. The event will be assessed by the local Principal Investigator and the form will be kept in the site file. Serious adverse events will be reported in the periodic safety reports to the research ethics committee and Trial Steering committee.

All adverse events (serious or other based on the definitions above) will be recorded on the case report form and details will be **entered on the study database within 1 week of completing the paper form**. Any SAEs which are deemed related to the trial intervention, the site will complete the paper CRF and **fax details this form to the CTRU within 24 hours of becoming aware of the event** in order for the CTRU to report this event to the Sponsor and the main REC within the required timeframes (15 days).

In participants using the Bi-Neb, any Adverse or SAEs relating to the use of Promixin via this device will be reported to the Patient Support team (PSP) at Phillips as per their standard practice.

12.4 Auditing

The sponsor will permit monitoring and audits by the relevant authorities, including the Research Ethics Committee. The investigator will also allow monitoring and audits by these bodies and the sponsor, and they will provide direct access to source data and documents.

12.5 Finance and indemnity

The trial has been financed by the NIHR and details have been drawn up in a separate agreement. This is an NHS sponsored study. If there is negligent harm during the clinical

trial when the NHS body owes a duty of care to the person harmed, NHS Indemnity will cover NHS staff, medical academic staff with honorary contracts and those conducting the trial. NHS Indemnity does not offer no-fault compensation and is unable to agree in advance to pay compensation for non-negligent harm. Ex-gratia payments may be considered in the case of a claim.

13. Ethics and dissemination

13.1 Approvals

The trial will be conducted subject to Research Ethics Committee favourable opinion including any provisions for site specific assessment. The application will be submitted through the IRAS central allocation system. The approval letter from the ethics committee and copy of approved patient information leaflets, consent forms and any ethically approved questionnaires will be present in the site files before initiation of the study and patient recruitment. Local research governance approvals will be sought from all participating research sites. This clinical trial will be conducted in accordance with Good Clinical Practice Guidelines and CTRU standard operating procedures. MHRA approval is not required for this study.

13.2 Protocol amendments

The investigator will be updated following an amendment to the protocol or study documents. The new documents, REC approval, R&D approval, HRA assessment letter and any other appropriate documentation surrounding the amendment will be sent to the site via a "site file update". The sites will receive the documents with a site file update sheet, detailing where to file the amended documents and which documents to supersede. If there are any significant changes to the study procedures or eligibility criteria sites will be notified by a combination of email, telephone, newsletters or additional project training when required.

In relation to informing REC, if any study documents require amending, the changes will be discussed with the sponsor and either a substantial (via IRAS and HRA) or minor amendment (notification via email) will be submitted to REC and HRA. Following REC acknowledgment and approval (when applicable) other appropriate approvals will be obtained i.e. HRA and R&D approval.

If a protocol amendment requires participants to be re-consented they will be informed of the amendment by an updated participant information sheet and will be asked to re-consent to the study. Trial registries, journals and regulators will be updated regarding protocol amendments when appropriate.

13.3 Consent

Consent for the main trial:

The ACtiF trial interventionist or local PI at the site will be responsible for taking informed consent from potentially eligible trial participants face to face at home or in clinic. Any researcher or clinical member of the team taking informed consent will be trained in study procedures and GCP. Participants will have the option to specify whether they are

interested in being approached for the qualitative interviews and audio recordings. However, they do not have to consent to these to be involved in the main study.

Consent for the interviews:
Consent for interviews (participant, interventionist or MDT member) will separately be taken by the qualitative researcher. Participants can participate in the main trial but choose to not take part in the qualitative research.

13.4 Confidentiality

Participant confidentiality will be respected at all times. Participant names and contact details will be collected and entered on the prospect database. Access to these personal details will be restricted to users with appropriate privileges only. All users who do not require access to identifiable data will only identify data by participant ID number, and no patient identifiable data will be transferred from the database to the statistician.

Trial documents (paper and electronic) will be retained in a secure location during and after the trial has finished. All source documents will be retained for a period of 5 years following the end of the trial. Where trial related information is documented in the medical records – those records will be retained for 5 years after the last patient last visit. Each site is responsible for ensuring records are archived and the information supplied to the Chief Investigator.

Any participant data held within CFHealthHub will be stored on a secure server at the University of Manchester. CFHealthHub complies with the Data Protection Act and follows best practice guidelines on security and information governance. Encrypted channels are used to transfer any data to and from the web and mobile application platforms. All user interaction with the CFHealthHub server and each action performed by a user will be logged. An audit log contains the username of the user performing the action, the date & time of the action, short description of the action performed. All users are authenticated via a secure password a with access to the system restricted on a role basis.

13.5 Declaration of Interests

Martin Wildman has received funding from Zambon who market the Ineb to carry out research to understand the performance of the Ineb and in the past we received funding from Zambon to carry out work to understand barriers to adherence.

13.6 Access to data

The central ACtiF study team alone will have access to the final dataset details of which will be outlined in the study DMP.

13.7 Ancilliary and post-trial care

Centres will be able to continue to use CFHealthHub if they wish to do so after the end of the pilot and feasibility study. If so, participants in the control arm will be able to cross over to use the intervention at this stage.

13.8 Dissemination policy

As this is a feasibility study its main interest will be to potential researchers and funding bodies. Data will be reported according to the revised CONSORT statement (Schultz,

2010). The findings of this research will be available to NIHR, patient groups and other interested bodies. It will also be offered for presentation at medical meetings and will be offered for publication in peer reviewed medical journals.

References

1. Alexander BM, Petren EK, Grimes M, Fink A, Myers V, Sewall A: **Mission of the cystic fibrosis foundation Annual Data Report 2013 Cystic Fibrosis Foundation Patient Registry**. 2014.

2. Southern KW, Barker PM, Solis-Moya A, Patel L: **Macrolide antibiotics for cystic fibrosis**. In *Cochrane Database of Systematic Reviews*. Edited by Southern KW. Chichester, UK: John Wiley & Sons, Ltd; 2011.

3. Ramsey BW, Davies J, McElvaney NG, Tullis E, Bell SC, Dřevínek P, Griese M, McKone EF, Wainwright CE, Konstan MW, Moss R, Ratjen F, Sermet-Gaudelus I, Rowe SM, Dong Q, Rodriguez S, Yen K, Ordoñez C, Elborn JS: **A CFTR Potentiator in Patients with Cystic Fibrosis and the *G551D* Mutation**. *N Engl J Med* 2011, **365**:1663–1672.

4. McCoy KS, Quittner AL, Oermann CM, Gibson RL, Retsch-Bogart GZ, Montgomery AB: **Inhaled aztreonam lysine for chronic airway Pseudomonas aeruginosa in cystic fibrosis.** *Am J Respir Crit Care Med* 2008, **178**:921–8.

5. Ryan G, Mukhopadhyay S, Singh M: **Nebulised anti-pseudomonal antibiotics for cystic fibrosis**. In *Cochrane Database of Systematic Reviews*. Edited by Ryan G. Chichester, UK: John Wiley & Sons, Ltd; 2003.

6. Jones AP, Wallis C: **Dornase alfa for cystic fibrosis**. In *Cochrane Database of Systematic Reviews*. Edited by Jones AP. Chichester, UK: John Wiley & Sons, Ltd; 2010.

7. Wark P, McDonald VM: **Nebulised hypertonic saline for cystic fibrosis**. In *Cochrane Database of Systematic Reviews*. Edited by Wark P. Chichester, UK: John Wiley & Sons, Ltd; 2009.

8. Ryan G, Singh M, Dwan K: **Inhaled antibiotics for long-term therapy in cystic fibrosis**. In *Cochrane Database of Systematic Reviews*. Edited by Ryan G. Chichester, UK: John Wiley & Sons, Ltd; 2011.

9. Eakin MN, Bilderback A, Boyle MP, Mogayzel PJ, Riekert KA: **Longitudinal association between medication adherence and lung health in people with cystic fibrosis**. *J Cyst Fibros* 2011, **10**:258.

10. Briesacher BA, Quittner AL, Saiman L, Sacco P, Fouayzi H, Quittell LM, Accurso F, Modi A, Lim C, Yu N, Geller D, Wagner M, Quittner A, DiMatteo M, Quittner A, Barker D, Marciel K, Grimley M, Emerson J, Rosenfeld M, McNamara S, Ramsey B, Gibson R, Nixon G, Armstrong D, Carzino R, Carlin J, Olinsky A, Robertson C, Grimwood K, et al.: **Adherence with tobramycin inhaled solution and health care utilization**. *BMC Pulm Med* 2011, **11**:5.

11. AL Q, KA R, Zhang J: **Relationship between adherence to pulmonary medications and health care costs: longitudinal analyses from 2005-2011.** *Pediatr Pulmonol* 2012, **47**:198.

12. Smyth A, Lewis S, Bertenshaw C, Choonara I, McGaw J, Watson A: **Case-control study of acute renal failure in patients with cystic fibrosis in the UK.** *Thorax* 2008, **63**:532–5.

13. McManus P: *Lead Pharmacist - Specialised Services (South Yorkshire and Bassetlaw) NHS England*. .

14. Daniels T, Goodacre L, Sutton C, Pollard K, Conway S, Peckham D: **Accurate assessment of adherence: self-report and clinician report vs electronic monitoring of nebulizers.** *Chest* 2011, **140**:425–32.

15. McNeil S: *Personal Communication from Stephanie McNeil, Chief Statistician of UK CF Registry*. .

16. Horne RW, Barber N, Elliot R, Morgan M: *Concordance, Adherance and Compliance in Medicine Taking: A Conceptual Map and Research Priorities. National Co-Ordinating Centre for NHS Service Delivery and Organisational R&D (NCCSDO)*. 2005.

17. Craig P, Dieppe P, Macintyre S, Michie S, Nazareth I, Petticrew M: **Developing and evaluating complex interventions: the new Medical Research Council guidance**. *BMJ* 2008:a1655–a1655.

18. Michie S, van Stralen MM, West R: **The behaviour change wheel: a new method for characterising and designing behaviour change interventions.** *Implement Sci* 2011, **6**:42.

19. Parham R, Thomas S, Mills R, Al. E: **CF patient's beliefs about nebuliser treatment: implications for adherence to treatment**. *J Cyst Fibros* 2012, **11**(Suppl 1):S9.

20. Bucks RS, Hawkins K, Skinner TC, Horn S, Seddon P, Horne R: **Adherence to treatment in adolescents with cystic fibrosis: the role of illness perceptions and treatment beliefs.** *J Pediatr Psychol* 2009, **34**:893–902.

21. George M, Rand-Giovannetti D, Eakin MN, Borrelli B, Zettler M, Riekert KA: **Perceptions of barriers and facilitators: self-management decisions by older adolescents and adults with CF.** *J Cyst Fibros* 2010, **9**:425–32.

22. Dziuban EJ, Saab-Abazeed L, Chaudhry SR, Streetman DS, Nasr SZ: **Identifying barriers to treatment adherence and related attitudinal patterns in adolescents with cystic fibrosis.** *Pediatr Pulmonol* 2010, **45**:450–8.

23. Bregnballe V, Schiøtz PO, Boisen KA, Pressler T, Thastum M: **Barriers to adherence in adolescents and young adults with cystic fibrosis: a questionnaire study in young patients and their parents.** *Patient Prefer Adherence* 2011, **5**:507–15.

24. Modi AC, Quittner AL: **Barriers to treatment adherence for children with cystic fibrosis and asthma: What gets in the way?** *J Pediatr Psychol* 2006, **31**:846–858.

25. Abbott J, Dodd M, Bilton D, Webb AK: **Treatment compliance in adults with cystic fibrosis.** *Thorax* 1994, **49**:115–20.

26. Abbott J, Dodd M, Webb AK: **Health perceptions and treatment adherence in adults with cystic fibrosis.** *Thorax* 1996, **51**:1233–8.

27. Abbott J, Dodd M, Gee L, Webb K: **Ways of coping with cystic fibrosis: implications for treatment adherence.** *Disabil Rehabil* 2001, **23**:315–24.

28. Conway SP, Pond MN, Hamnett T, Watson A: **Compliance with treatment in adult patients with cystic fibrosis.** *Thorax* 1996, **51**:29–33.

29. Jones S, Curley R, Wildman M: **345 Systematic review of qualitative studies investigating barriers to adherence in patients with cystic fibrosis using framework analysis structured by a conceptual framework of behaviour change**. *J Cyst Fibros* 2013, **12**(Suppl 1):S136.

30. Kettler LJ, Sawyer SM, Winefield HR, Greville HW: **Determinants of adherence in adults with cystic fibrosis.** *Thorax* 2002, **57**:459–64.

31. Lask B: **Non-Adherence to Treatment in Cystic Fibrosis**. *J R Soc Med* 1994, **87**(21 Suppl):25–27.

32. Latchford G, Duff A, Quinn J, Conway S, Conner M: **Adherence to nebulised antibiotics in cystic fibrosis.** *Patient Educ Couns* 2009, **75**:141–4.

33. Horne R, Weinman J, Barber N, Elliott R: *Concordance , Adherence and Compliance in Medicine Taking: Report for the National Co-Ordinating Centre for NHS Service Delivery and Organisation R & D (NCCSDO)*. London: NCCSDO; 2005.

34. Ivers N, Jamtvedt G, Flottorp S, Young JM, Odgaard-Jensen J, French SD, O'Brien MA, Johansen M, Grimshaw J, Oxman AD: **Audit and feedback: effects on professional practice and healthcare outcomes.** *Cochrane database Syst Rev* 2012, **6**:CD000259.

35. Geraedts H, Zijlstra A, Bulstra SK, Stevens M, Zijlstra W: **Effects of remote feedback in home-based physical activity interventions for older adults: a systematic review.** *Patient Educ Couns* 2013, **91**:14–24.

36. Cane J, O'Connor D, Michie S: **Validation of the theoretical domains framework for use in behaviour change and implementation research.** *Implement Sci* 2012, **7**:37.

37. Thabane L, Ma J, Chu R, Cheng J, Ismaila A, Rios LP, Robson R, Thabane M, Giangregorio L, Goldsmith CH: **A tutorial on pilot studies: the what, why and how.** *BMC Med Res Methodol* 2010, **10**:1.

38. Baranowski T, Stables G: **Process Evaluations of the 5-a-Day Projects**. *Heal Educ Behav* 2000, **27**:157–166.

39. Pfadenhauer LM, Mozygemba K, Gerhardus A, Hofmann B, Booth A, Lysdahl KB, Tummers M, Burns J, Rehfuess EA: **Context and implementation: A concept analysis towards conceptual maturity**. *Z Evid Fortbild Qual Gesundhwes* 2015, **109**:103–114.

40. Weber K, Glynn M a.: **Making Sense with Institutions: Context, Thought and Action in Karl Weick's Theory**. *Organ Stud* 2006, **27**:1639–1660.

41. Hyatt D: **Time for a change: a critical discoursal analysis of synchronic context with diachronic relevance**. *Discourse Soc* 2005, **16**:515–534.

42. Linnan L, Steckler A: **Process evaluation for public health interventions and research: an overview**. In *Process evaluation for public health interventions and research*. 1st edition. Edited by Linnan L, Steckler A. San Francisco: Jossey-Bass; 2002:1–23.

43. Moore GF, Audrey S, Barker M, Bond L, Bonell C, Hardeman W, Moore L, O'Cathain A, Tinati T, Wight D, Baird J: **Process evaluation of complex interventions: Medical Research Council guidance.** *BMJ* 2015, **350**:h1258.

44. Nilsen P: **Making sense of implementation theories, models and frameworks**. *Implement Sci* 2015, **10**:1–13.

45. Meyers DC, Durlak J a., Wandersman A: **The Quality Implementation Framework: A Synthesis of Critical Steps in the Implementation Process**. *Am J Community Psychol* 2012, **50**:462–480.

46. Borrelli B: **The Assessment, Monitoring, and Enhancement of Treatment Fidelity In Public Health Clinical Trials.** *J Public Health Dent* 2011, **71**:S52–S63.

47. Fuchs HJ, Borowitz DS, Christiansen DH, Morris EM, Nash ML, Ramsey BW, Rosenstein BJ, Smith AL, Wohl ME: **Effect of aerosolized recombinant human DNase on exacerbations of respiratory symptoms and on pulmonary function in patients with cystic fibrosis. The Pulmozyme Study Group.** *N Engl J Med* 1994, **331**:637–42.

48. Ratjen F, Durham T, Navratil T, Schaberg A, Accurso FJ, Wainwright C, Barnes M, Moss RB: **Long term effects of denufosol tetrasodium in patients with cystic fibrosis.** *J Cyst Fibros* 2012, **11**:539–49.

49. Miller MR, Hankinson J, Brusasco V, Burgos F, Casaburi R, Coates A, Crapo R, Enright P, van der Grinten CPM, Gustafsson P, Jensen R, Johnson DC, MacIntyre N, McKay R, Navajas D, Pedersen OF, Pellegrino R, Viegi G, Wanger J: **Standardisation of spirometry.** *Eur Respir J* 2005, **26**:319–38.

50. Herdman M, Gudex C, Lloyd A, Janssen M, Kind P, Parkin D, Bonsel G, Badia X: **Development and preliminary testing of the new five-level version of EQ-5D (EQ-5D-5L).** *Qual Life Res* 2011, **20**:1727–36.

51. Hibbard JH, Mahoney ER, Stockard J, Tusler M: **Development and testing of a short form of the patient activation measure.** *Health Serv Res* 2005, **40**(6 Pt 1):1918–30.

52. Wong MD, Sarkisian C a., Davis C, Kinsler J, Cunningham WE: **The Association Between Life Chaos, Health Care Use, and Health Status Among HIV-Infected Persons**. *J Gen Intern Med* 2007, **22**:1286–1291.

53. Verplanken B, Orbell S: **Reflections on Past Behavior: A Self-Report Index of Habit Strength1**. *J Appl Soc Psychol* 2003, **33**:1313–1330.

54. Quittner a L, Sweeny S, Watrous M, Munzenberger P, Bearss K, Gibson Nitza a, Fisher L a, Henry B: **Translation and linguistic validation of a disease-specific quality of life measure for cystic fibrosis.** *J Pediatr Psychol* 2000, **25**:403–14.

55. Kroenke K, Strine TW, Spitzer RL, Williams JBW, Berry JT, Mokdad AH: **The PHQ-8 as a measure of current depression in the general population**. *J Affect Disord* 2009, **114**:163–173.

56. Spitzer RL, Kroenke K, Williams JBW, Löwe B: **A brief measure for assessing generalized anxiety disorder: the GAD-7.** *Arch Intern Med* 2006, **166**:1092–7.

57. Horne R, Weinman J, Hankins M: **The beliefs about medicines questionnaire: The development and evaluation of a new method for assessing the cognitive representation of medication**. *Psychol Health* 1999, **14**:1–24.

58. Lancaster GA, Dodd S, Williamson PR: **Design and analysis of pilot studies:**

recommendations for good practice. *J Eval Clin Pract* 2004, **10**:307–12.

59. Arain M, Campbell MJ, Cooper CL, Lancaster GA: **What is a pilot or feasibility study? A review of current practice and editorial policy.** *BMC Med Res Methodol* 2010, **10**:67.

60. Eldridge S, Bond C, Campbell M, Lancaster G, Thabane L, Hopwell S: **Definition and reporting of pilot and feasibility studies**. *Trials* 2013, **14**(Suppl 1):O18.

61. Schulz KF, Altman DG, Moher D: **CONSORT 2010 statement: Updated guidelines for reporting parallel group randomized trials**. *Annu Intern Med* 2010, **152**:726–732.

62. Ritchie J, Spencer L: **Qualitative data analysis for applied policy research**. In *Analysing qualitative data*. Edited by Bryman A, Burgess RG. Routledge; 1994:173–194.

63. Horne R: **Treatment perceptions and self-regulation**. In *The Self-regulation of Health and Illness Behaviour*. Edited by Cameron L, Leventhal H. London: Routledge; 2003:138–53.

64. Moore GF, Audrey S, Barker M, Bond L, Bonell C, Hardeman W, Moore L, O'Cathain A, Tinati T, Wight D, Baird J: **Process evaluation of complex interventions: Medical Research Council guidance**. *BMJ* 2015, **350**(mar19 6):h1258–h1258.

65. Farmer T, Robinson K, Elliott SJ, Eyles J: **Developing and implementing a triangulation protocol for qualitative health research.** *Qual Health Res* 2006, **16**:377–94.

66. O'Cathain A, Murphy E, Nicholl J, Cathain AO: **Three techniques for integrating data in mixed methods studies**. *BMJ* 2010, **341**(sep17 1):c4587–c4587.

Appendix 1. W.H.O. Trial Registration Data Set

DATA CATEGORY	INFORMATION
Primary registry and trial identifying number	To be added
Date of registration in primary registry	To be added
Secondary identifying numbers	NIHR: RP-PG-1212-20015<br>Sponsor (STH): STH19213
Source(s) of monetary or material support	National Institute for Health Research (NIHR) Programme Grants for Applied Research programme.
Primary sponsor	Sheffield Teaching Hospitals NHS Foundation Trust.
Secondary sponsor(s)	none
Contact for public queries	Chin Maguire<br>Trial Manager<br>Clinical Trials Research Unit<br>University of Sheffield<br>Regent Court<br>30 Regent Street<br>Sheffield<br>S1 4DA<br>Tel: (+44) (0)114 222 0717<br>Fax: (+44) (0)114 222 0870<br>email : c.maguire@sheffield.ac.uk
Contact for scientific queries	Dr Martin Wildman<br>Adult CF Centre<br>Northern General Hospital<br>Herries Road<br>Sheffield<br>S5 7AU<br>Tel: (0114) 2715212<br>Fax: (0114) 222 0870<br>email : Martin.Wildman@sth.nhs.uk
Public title	Adherence to treatment in adults with Cystic Fibrosis (ACtiF)
Scientific title	Development and evaluation of an intervention to support Adherence to treatment in adults with Cystic Fibrosis : a feasibility study comprised of an external pilot randomised controlled trial and process evaluation
Countries of recruitment	United Kingdom
Health condition(s) or problem(s) studied	Cystic Fibrosis
Intervention(s)	Usual care plus a microchipped nebuliser with or without a complex intervention. The complex intervention consists of:

	- A software platform, CFHealthHub mobile apps and website, which allows access to medication adherence data and education modules intended to remove barriers to adherence<br>- A manual containing a ‘behaviour change toolkit’ to guide interactions between health
Key inclusion and exclusion criteria	Inclusion criteria for participants<br>1.Diagnosed with CF and with data within the CF registry<br>2.Aged 16 years and above<br>3.Taking inhaled mucolytics or antibiotics via a chipped nebuliser (e.g. eTrack or Bi-Neb) or able and willing to take via eTrack or Bi-Neb.<br><br>Exclusion criteria for participants<br>1.Post-lung transplant<br>2.People on the active lung transplant list<br>3.Patients receiving palliative care,<br>4.Lacking in capacity to give informed consent<br>5.Using dry powder devices to take antibiotics or mucolytics
Study type	Feasibility study comprised of an external pilot randomised controlled trial and process evaluation
Date of first enrolment	Anticipated: 02/05/2016
Target sample size	We propose to recruit to time, that is for a fixed period of four months rather than to a fixed sample size. To match the proposed recruitment rate of the main RCT, the target sample over the four months for which the pilot RCT is open, will be n=64.
Recruitment status	Not yet open.
Primary outcome(s)	Exacerbations of cystic fibrosis as defined by the Fuchs criteria (*N Engl J Med* 1994, 331:637–42.)
Key secondary outcomes	None.

ACtiF Pilot Study

Control Patient Topic guide

Thank you for agreeing to take part in the interview today

Check timing ok

Check consent form filled in / received

Are you happy for the interview to be recorded?

We're interested in your experiences of the service that you receive for helping you to use your nebuliser.

1. Why did you decide to take part in the research?

2. How did you find being asked to take part in the trial? [Prompts: paperwork volume, information provided, questionnaires]

Now I'd like to ask you about the care you received before the trial started to help you use your nebuliser.

3. What types of things did the unit/hospital recommend that you do to help you use your nebuliser? [Prompts: appointments / what do you talk about? / nebulisers / skills to use your nebuliser properly / knowledge and beliefs?]

4. What types of things did the unit/hospital recommend that you do to help you use your nebuliser as much as possible? [Prompts: setting goals, solving problems, making plans, giving you information, building skills, beliefs about nebuliser medication, giving you confidence]

5. How did the care you received to help you use your nebuliser fit with any other care you received for CF more generally?

6. How could the care you received for helping you to use your nebuliser as prescribed be improved?

7. Overall how happy are you with the care you received for your nebuliser? [Prompts: what could be done better?]

Now I want to ask you about specific kinds of things that might have changed since the trial started:

8. Since you joined the trial has the care that you receive in the unit / hospital changed at all? [Prompts: Has anybody done anything different? What have they done?]

9. Since you joined the trial has anyone asked you to change how you use your nebuliser? If so, what have they suggested you do? [Prompt: capability skills / knowledge including beliefs / where has the change come from?]

10. Since you joined the trial has anyone suggested ways to help you use your nebuliser as much as possible? If so what? [Prompt: opportunity finding time to use nebuliser / making plans / setting goals / where has the change come from?]

11. Since you joined the trial has anyone helped you have more confidence to use your nebuliser as prescribed? [Prompt: where has the change come from? what have they done?]

Is there anything else that we haven't talked about that you'd like to comment on?

THANK YOU

The ACtiF Project is funded by the National Institute for Health Research's Programme Grants for Applied Research.

ACtiF Pilot Study

Intervention Patient Topic guide

Thank you for agreeing to take part in the interview today

Check timing ok

Check consent form filled in / received

Are you happy for the interview to be recorded?

We're interested in your experiences of the service that you have received from CFHealthHub including both the meetings to discuss your nebuliser medication and the website/app you have used.

1. Why did you decide to take part in the research?

2. How did you find being asked to take part in the trial? [Prompts: recruitment, paperwork volume, information provided, questionnaires]

Now I'd like to ask you about your meetings with the person who has been working with you on CFHealthHub.

3. What types of things did they recommend that you do? [prompts: setting goals, solving problems, making plans (myplan), giving you information]

4. Do you think you have had any benefit from these meetings?

    If yes, what benefit and what about the service helped you to get this?

    If no, what has stopped you gaining benefit?

5. What was good about how the meetings were delivered? [Prompt: what needs to be improved?]

Now I'd like to talk to you about the CFHealthHub website / app.

6. What was good about the website? [Prompts: my plan, how am I doing, tool kit, graphs, my treatment]

7. What needs to be improved? [Prompts: my plan, how am I doing, tool kit, graphs, my treatment]

8. Do you think you've had any benefit from using the website?

   If yes, what benefit and what about the website helped you to get this?

   If no, what has stopped you gaining benefit?

9. Have the website and/or meetings helped you to improve how often you use your nebuliser?

   If yes, how has it helped you to do this?

   If not, why not?

10. How do the CFHealthHub website and the meetings work together?

11. Has using CFHealthHub helped you to be able to use your nebuliser any better? Why / Why not? [Prompt: capability skills / knowledge including beliefs]

12. Has using CFHealthHub helped you to find the time to use your nebuliser more? Why / why not? [Prompt: opportunity / making plans]

13. Has using CFHealthHub made you want to use your nebuliser more? Why / why not? [Prompt: motivation and confidence]

14. How does the CFHealthHub service (website and meetings) fit with the care you were already receiving at the unit/hospital?

15. Do you think you would continue using CFHealthHub? [Prompt: during the study / after the study]

16. Is CFHealthHub a good thing to use in general for people with CF? Why? / Why not?

17. How have you found being part of the study?

Is there anything else that we haven't talked about that you'd like to comment on?

THANK YOU

The ACtiF Project is funded by the National Institute for Health Research's Programme Grants for Applied Research.

ACTIF Pilot Study

Interventionist Topic guide

Thank you for agreeing to take part in the interview today.

Check timing ok

Check consent form filled in / received

Are you happy for the interview to be recorded?

Introduction to the interview: Interested in how you've found using CFHealthhub (CFHH) with your participants and any learning from it

The trial

1. What works or could be improved about:
   a) recruiting patients to the trial?
   b) collecting data?
   c) any other aspect?

The intervention:

Now I'd like to go through each of the steps for providing the intervention to get your views on each of these

2. What works or could be improved about:
   a) how you have assessed participants' adherence levels prior to using CFHH?

   b) how you set up appointments with your participants?

   c) session 1? [Prompts: gathering data, introducing the nebuliser, entering prescription data into CFHH, completion of screening tools, patient feedback, anything else]

d) session 2? [Prompts: reviewing adherence data, introducing CFHH, explaining modules, setting goals, action planning, identifying suitable tailored content, technical issues, anything else]

e) session 3? [Prompts: reviewing goals, reviewing adherence plans, motivation, problem solving, anything else]

3. What works or could be improved about the training manuals and training sessions?

4. What works or could be improved about the support available from the research team? [Prompts: timing, availability, problem solving].
[**Specific prompt for MDT senior interventionist**: do you think the training has equipped you to deliver this intervention in your centre yourself after the trial ends? If no, what further training would be needed?]

5. How has the CFHH intervention been received by the rest of the team? [Prompt: how has your communication been with the rest of the team about CFHH?]

6. What sort of follow-up did participants request? How will you handle this?

7. How has the CFHH intervention helped your participants to know how to use their nebuliser? [Prompt: capability / skills, knowledge and beliefs]

8. How has the CFHH intervention helped your participants find ways to use their nebuliser more? [Prompt: opportunity]

9. How has the CFHH intervention helped to motivate your participants to use their nebuliser? [Prompt: motivation / confidence]

General questions:

10. How engaged did participants seem with CFHH? [Prompt: What feedback if any have you had from participants about CFHH?]

11. How useful do you think CFHH is for your participants?

12. How easy / difficult has it been to get your participants to use CFHH?

13. Have you seen any changes to the ways in which your participants use their nebulisers since starting CFHH?

14. What have you learnt from using CFHH with your participants?

15. What if any are the benefits to you and / or to your participants of using CFHH?

16. How do you think CFHH fits with the other care offered by the centre?

17. How have you found being part of the trial?

Is there anything else you'd like to say about CFHH?

THANK YOU

The ACtiF Project is funded by the National Institute for Health Research's Programme Grants for Applied Research.

ACTIF Pilot Study

MDT Topic guide

Thank you for agreeing to take part in the interview today.

Check timing ok

Check consent form filled in / received

Are you happy for the interview to be recorded?

We're interested in your views of the CFHealthHub service and how it fits into the care provided in your centre.

1. Can you describe the key things you did in your centre to help patients adhere to their nebulisers prior to the ACtiF study?

2. How does nebuliser adherence fit with the other things you do for CF patients?

3. What involvement have you had in the CFHealthHub intervention? [Prompts: website, interventionist, training of staff]

4. You had training to help you be more aware of patient activation. What did you think of the training? [Prompts: Do you think it has changed your practice in any way? If yes what changes, if no why not? Key aspects – patient knowledge including beliefs / skills / confidence]

5. Do you think CFHealthHub is a useful intervention? Why? / Why not? [Prompts: what do you think about the: website, feedback about adherence data, interventionist, training?]

6. How do you think the CFHealthHub intervention is operating in practice? [Prompt: what are the strengths / improvements needed?]

7. How does the CFHealthHub intervention fit with the care offered by your centre?

8. How does the CFHealthHub intervention help your patients to know how to use their nebuliser? [Prompts: Skills / knowledge / beliefs. How / Why doesn't it help?]

9. How does the CFHealthHub intervention help your patients to find ways to use their nebuliser more? [Prompts: How / Why doesn't it help?]

10. How does the CFHealthHub intervention help to motivate your patients to use their nebuliser? [Prompts: How / Why doesn't it help?]

11. Do you think CFHealthHub is helping your intervention patients to improve their adherence? If yes, what key things have helped this? If no, what if anything could be done to help this?

12. Has the CFHealthHub intervention changed anything about the way in which you and/or your team approach adherence in your centre?

i) for patients receiving the intervention?

ii) for patients not receiving the intervention?

[Prompts: MDT discussions / differences between control and intervention patients]

13. Which patient groups are most likely to benefit from CFHealthHub? Why?

14. Which patient groups are least likely to benefit from CFHealthHub? Why?

15. Would you consider continuing to use CFHealthHub in the future? Why? Why not?

16. How has it been for you / your centre taking part in the trial? [Prompt: recruitment to the study]

17. How able do you feel to go on delivering care related to improving adherence after the study ends? [Prompt: has the study changed the way you will go about this?]

18. Are there any aspects of the research that we haven't talked about that you'd like to comment on?

Is there anything else you'd like to say?

THANK YOU

The ACtiF Project is funded by the National Institute for Health Research's Programme Grants for Applied Research.
