# Supplementary material for: Supporting medication adherence for adults with cystic fibrosis: a randomised feasibility study
Source: BMC Pulm Med. 2019 Apr 11;19:77. doi: 10.1186/s12890-019-0834-6 (PMC6458785; doi:10.1186/s12890-019-0834-6)
Supplement: Supplementary file 3 — Release notes for 59 software development cycles between June 2015 and June 2017. (DOCX 17 kb) [file 12890_2019_834_MOESM3_ESM.docx]

**Additional File 1 - Release notes for 59 software development cycles between June 2015 and June 2017**

| **No.** | **Date** | **Release Notes** |
| --- | --- | --- |
| 1 | 03-Jun-15 | First test release to try out the Release SOP |
| 2 | 17-Jun-15 | First release for the development server |
| 3 | 01-Aug-15 | First release - testing for 5 patient trial |
| 4 | 06-Aug-15 | Release for 5 patient trial |
| 5 | 12-Oct-15 | BCI Release - includes education, problem solving and toolkit - allows clinicians to add/edit patients - patient analytics included |
| 6 | 23-Oct-15 | BCI Minor updates release - added missing Colistin information to Problem Solving - fixed graph resize issue - integrated design fixes |
| 7 | 01-Dec-15 | Iterative BCI release - Action plans - Graph tweaks to use traffic light system and show treatment times in labels - Added analytics to the graph viewing |
| 8 | 03-Dec-15 | Iterative BCI Release - minor bug fixes to graph to show target line >95% - minor link fixes to Problem-Solving and My Toolkit |
| 9 | 15-Dec-15 | Iterative BCI Release - Updated Design styles for My Treatment - 24 hour clock used in tooltip labels of daily graphs |
| 10 | 18-Dec-15 | Iterative BCI Release - minor fix for broken video link and My Treatment arrow icons |
| 11 | 11-Feb-16 | Iterative BCI Release - Clinician Report Page Added - Screening Tool Added - Coping Plans Added - Revised Design and Content of My Treatment - Revised Design and Content of Problem Solving - Revised Design and Content of My Toolkit |
| 12 | 19-Feb-16 | First Mobile App Release - Mobile app Release - Fix for toolkit issue in patient view - Fix for Aztreonam (twice updated to three times daily in My Treatment) |
| 13 | 22-Feb-16 | - Mini updates to the mobile app - Fix for array handling |
| 14 | 25-Feb-16 | - Fix for My Treatment bug in mobile app - Changes to screening tool |
| 15 | 09-Apr-16 | - Added Day and Party Planners  - New home page for web and mobile apps  - Videos added  - Data sharing option with MDT added  - Added support for control group  - Added support for researcher role  - Added ALL setting to centres |
| 16 | 26-Apr-16 | - Added consent page  - Updated prescription flow  - Updated How am I Doing designs  - Remove reference to prescription guideline in adherence flow  - Enhanced validation on add/edit patient  - Updated party planner activities  - Support for changing wallpaper  - New navigation menu integrated |
| 17 | 09-May-16 | - Included video support for multiple categories  - New batch of participant videos  - Layout and design updates to My Treatment and Problem Solving  - Added support for push notifications  - Updates to layout of clinician patient page  - Store history of alerts |
| 18 | 10-May-16 | Minor update to fix DB issue |
| 19 | 10-May-16 | DB upgrade fixes |
| 20 | 13-May-16 | - Fix for menu item not showing Day and Party Planners in Patient View  - Fix for clinician analytics  - Update to notification wording in the patient settings menu |
| 21 | 22-Jun-16 | - Optimisation of website  - Screening Tool Review feature added  - New homepage added  - New videos added  - Various bug fixes |
| 22 | 26-Jun-16 | ActiF Pilot release version |
| 23 | 29-Jun-16 | - Minor bug fixes to adherence view for existing patients  - Minor bug fix to the screening tool for thread-safety |
| 24 | 29-Jun-16 | Fix for ‘check progress’ links on control patients in prescription view of clinicians |
| 25 | 05-Jul-16 | Updates to the screening tool review feature:   - removed 0 scores,   - indicators on toolkit/problem solving items in the selection list  - minor updates to the history handling |
| 26 | 06-Jul-16 | Updates to the screening tool to show red colour for items in current toolkit and to fix menu screening tool bug. |
| 27 | 11-Jul-16 | Added missing question to screening tool |
| 28 | 11-Jul-16 | Fix for Screening Tool menu issue for researcher role |
| 29 | 21-Jul-16 | Renamed the rewards DB table. |
| 30 | 25-Jul-16 | Added instructions for installing and downloading the mobile app |
| 31 | 27-Jul-16 | Fix for problem with target adherence percentage |
| 32 | 27-Jul-16 | Liquibase fix for target adherence change |
| 33 | 04-Aug-16 | Add patient mobile phone details to patient screens |
| 34 | 04-Aug-16 | Bug fix for patient form |
| 35 | 12-Aug-16 | Small fixes in preparation for ios app release |
| 36 | 16-Aug-16 | Add ios push notifications support |
| 37 | 23-Aug-16 | - Bug fix for clinician analytics  - Bug fix for app settings  - Automation of android app build |
| 38 | 04-Oct-16 | - Support for Bi-neb device  - Preparing for iOS app release  - Some fixes on toolkit pages |
| 39 | 01-Nov-16 | - support for export feature - fix for DST/UTC issue with Bineb device - Workaround for graph/table no-show issue |
| 40 | 07-Nov-16 | - First draft implementation of data observatory landing page - Fix for export feature; table name case issue - Remove of DST/UTC custom handling |
| 41 | 20-Dec-16 | - Updated CSRF protection  - Detect duplicate entries in observation on import  - Library updates   - Various bug fixes  - Update to Jackson version 2.8.5   - Update to Angular version 1.6  - Added password strength indicators  - Fixed custom wallpaper issue  - Enhanced rate limiting for login   - Added ability to create export user  - Show historic target percentages  - Added line graph support |
| 42 | 20-Jan-17 | Fix adherence to handle patients with no target |
| 43 | 20-Jan-17 | Fix for video menu |
| 44 | 22-Jan-17 | Fix for password reset email link not working. Now specifies query param for the password reset url. |
| 45 | 22-Jan-17 | Pass new URL link for password reset |
| 46 | 13-Feb-17 | - Data observatory MDT View landing page  - Data observatory Meeting View  - Data observatory entry fields for DOB, weight, height, pseudomonas status  - Bineb partial nebulisation release |
| 47 | 14-Feb-17 | - Added date of birth support to MDT landing page  - Changed ‘DOB’ to ’Date of birth’  - Added ‘add patient’ button to MDT landing page |
| 48 | 16-Feb-17 | - Bug fixes  - Refactoring around client side patient add/edit controllers  - Added test build profile |
| 49 | 09-Mar-17 | - Updates to push notifications  - Duplicate handling  - New videos release |
| 50 | 10-Mar-17 | Added Summary Page |
| 51 | 13-Mar-17 | - Fix for editing control patients - Fix for screening tool not working in MDT View |
| 52 | 17-Mar-17 | Performance enhancements, removed redundant code. |
| 53 | 11-Apr-17 | - Release of the summary review page - Minor fix for line graph - Includes changes to fix adherence views in the mobile app - Fix for data observatory MDT sharing flag controls |
| 54 | 27-Apr-17 | - Reworked Bineb partial nebulisation (.5 for partial nebs and enlarged the * display) - Routine maintenance on adherence refactoring - Includes new consent text for data observatory vs RCT use - Name change to update from ‘MDT View’ to ‘Meeting View in table displays - Adherence export work to allow extraction of stats  - Fix for some videos not displaying correctly - Fix for summary log not saving when some fields not completed - Fix for summary review date to show date when summary was saved |
| 55 | 28-Apr-17 | - Fix for liquibase consent script - Fix for test data (mobile and home phone numbers had spaces in) |
| 56 | 17-May-17 | - Added new prescription flow to capturing alternating vs continuous regimes and free text notes - Added coping plans to reports - Added video transcripts - Shows green in table view when achieving historical targets |
| 57 | 26-May-17 | - Added case study data - Fix for export unit tests |
| 58 | 21-Jun-17 | - Updates to training case studies - Updated flow for ‘continuous’ drug - Add patient view access via clinician view - Formatting display fix for summary review page - Toolkit page export tweak |
| 59 | 22-Jun-17 | - Fixes for patient view from clinician view transition  - Updated BDD |
