# Supplementary material for: Supporting medication adherence for adults with cystic fibrosis: a randomised feasibility study
Source: BMC Pulm Med. 2019 Apr 11;19:77. doi: 10.1186/s12890-019-0834-6 (PMC6458785; doi:10.1186/s12890-019-0834-6)
Supplement: Supplementary file 5 — Changes to intervention and trial procedures. (DOCX 22 kb) [file 12890_2019_834_MOESM5_ESM.docx]

**Additional File 05. Changes to intervention and trial procedures drawn from the pilot RCT**

| **Change Number** | **Problem type** | **Problem identified** | **Solutions implemented in full-scale trial (# numbers refer to logic model constructs affected- see Figure 1)** |
| --- | --- | --- | --- |
| **CFHealthHub IT component** |  |  |  |
| 1 | Real world and trial | Clinician functionality (amending prescriptions/ treatment targets) inaccessible through participant view (used in intervention sessions). | Patient view accessibility enabled through clinician view (#3). |
| **Other IT infrastructure** |  |  |  |
| 2 | Real World and Trial | Flatlines in adherence graphs at the time of nebuliser pairing **(#4)** with Qualcomm Hub **(#5)**. | Quality assurance of adherence data maintained by pairing hardware at the factory (#4, #5, #14, #35). |
| **RCT procedures** |  |  |  |
| 3 | Trial | Participant dislike of repetition between PROMS | Interventionists trained to anticipate this and explain necessity of repetition. |
| 4 | Trial | Poor understanding of site definitions and differences in ‘usual care’ | Usual care questionnaire designed and administered at all sites. |
| 5 | Trial | Interventionist difficulty in categorisation of medications for data entry. Many did not have a CF clinical background/ knowledge. | Database entry forms amended so that categorisation is not necessary. |
| 6 | Trial | Study completion form difficult to complete. | Study completion form redesigned to simplify recording of withdrawals. |
| 7 | Trial | Exacerbations form difficult to complete. | Exacerbation form redesigned to allow recording of cases in which oral antibiotics prescribed for exacerbations. |
| 8 | Trial | Patient information sheet too long. | Research Ethics Committee requirements meant the sheet could not be amended. A short participant information leaflet was created and approved by REC for use in initial participant approach. |
| 9 | Trial | Pre-post analysis of adherence data lacked sensitivity to periods of success and higher adherence during the study (#35). | To capture this variation more effectively, the statistical analysis plan for the full-scale trial will include analyses to capture this. |
| 10 | Trial | Interventionist workload balance to meet recruitment targets and deliver intervention (#18, #24, #25, #26). | Recruitment period extended. Weekly interventionist calls to discuss intervention delivery targets and fidelity assessments have also been implemented. |
